# Unveiling the role of TGF-β signaling pathway in breast cancer prognosis and immunotherapy

**DOI:** 10.3389/fonc.2024.1488137

**Published:** 2024-11-27

**Authors:** Yifan Zheng, Li Li, Wenqian Cai, Lin Li, Rongxin Zhang, Wenbin Huang, Yulun Cao

**Affiliations:** ^1^ Department of General Surgery I, The First Affiliated Hospital of Guangdong Pharmaceutical University, Guangzhou, Guangdong, China; ^2^ Guangdong Provincial Key Laboratory for Biotechnology Drug Candidates, Institute of Basic Medical Sciences and Department of Biotechnology, School of Life Sciences and Biopharmaceutics, Guangdong Pharmaceutical University, Guangzhou, China; ^3^ Department of Hepatobiliary Surgery II, Zhujiang Hospital, Southern Medical University, Guangzhou, China

**Keywords:** breast cancer, TGF-β, prognostic signature, tumor microenvironment, immunotherapy

## Abstract

**Introduction:**

The TGF-β signaling pathway (TSP) is pivotal in tumor progression. Nonetheless, the connection between genes associated with the TSP and the clinical outcomes of breast cancer, as well as their impact on the tumor microenvironment and immunotherapeutic responses, remains elusive.

**Methods:**

Breast cancer transcriptomic and single-cell sequencing data were obtained from the The Cancer Genome Atlas (TCGA) and the Gene Expression Omnibus (GEO) databases. We identified 54 genes associated with the TSP from the Molecular Signatures Database (MSigDB) and analyzed both data types to evaluate TSP activity. Using weighted gene co-expression network analysis (WGCNA), we identified modules linked to TSP activity. To assess patient risk, we used 101 machine learning algorithms to develop an optimal TGF-β pathway-related prognostic signature (TSPRS). We then examined immune activity and response to immune checkpoint inhibitors and chemotherapy in these groups. Finally, we validated ZMAT3 expression levels clinically and confirmed its relevance in breast cancer using CCK-8 and migration assays.

**Results:**

At the single-cell level, TSP activity was most notable in endothelial cells, with higher activity in normal tissues compared to tumors. TSPRS was developed. This signature's accuracy was confirmed through internal and external validations. A nomogram incorporating the TSPRS was created to improve prediction accuracy. Further studies showed that breast cancer patients categorized as low-risk by the TSPRS had higher immune phenotype scores and more immune cell infiltration, leading to better prognosis and enhanced immunotherapy response. Additionally, a strong link was found between the TSPRS risk score and the effectiveness of anti-tumor agents. Silencing the ZMAT3 gene in the TSPRS significantly reduced the proliferation and invasiveness of breast cancer cells.

**Discussion:**

Our study developed a TSPRS, which emerges as a potent predictive instrument for the prognosis of breast cancer, offering novel perspectives on the immunotherapeutic approach to the disease.

## Introduction

Breast cancer, with its high incidence and mortality rates among women globally, stands as a leading cause of cancer-related deaths. According to the latest data from the International Agency for Research on Cancer in 2020, breast cancer has become the most prevalent cancer worldwide, posing a significant threat to women’s health (1). The complexity of breast cancer is further underscored by its classification into various subtypes such as luminal A, luminal B, HER2-positive, and triple-negative breast cancer (TNBC), each with distinct biological behaviors and treatment responses. Among these, TNBC is particularly challenging due to its high malignancy, lack of relevant targets, poor prognosis, and limited treatment options.

Although conventional treatments such as surgical resection, radiotherapy, chemotherapy, targeted therapy, and endocrine therapy have improved outcomes, they are not always successful, leading to a persistent search for more effective strategies. In this context, immunotherapy has emerged as a groundbreaking approach that has shown remarkable success in treating melanoma and lung cancer (2, 3). This success has paved the way for exploring the potential benefits of immunotherapy in breast cancer, traditionally considered to have “low immunogenicity”.

The pursuit of effective immunotherapy for breast cancer has concentrated on vaccines, Chimeric Antigen Receptor T cell (CAR-T) therapy, and immune checkpoint inhibitors (ICIs). ICIs, in particular, have become a focal point of clinical research. While monotherapy with ICIs has not been ideally successful, there is a growing interest in combination therapies. Despite the low immunogenicity attributed to breast cancer, its high heterogeneity presents both challenges and opportunities. TNBC, for example, is characterized by stronger immune infiltration and higher genomic instability compared to other subtypes (4), suggesting a better response to immunotherapy in certain patient populations. Meanwhile, hormone receptor-positive breast cancers, once deemed “cold” tumors, are now recognized to exhibit varying levels of immune activity (5, 6), indicating that targeted therapies could be beneficial for a subset of these patients.

The role of TGF-β, a multifunctional cytokine, in cancer progression illustrates the complexity of the tumor microenvironment (TME) in breast cancer. TGF-β can act as both a tumor suppressor and promoter, depending on the stage of the disease and the cellular context (7–9). Its dual role in the immune response further complicates the landscape, as it can inhibit anti-tumor immunity while promoting immune tolerance and tumor escape. Understanding the activity of the TGF-β signaling pathway (TSP) in the breast cancer TME is thus crucial for advancing immunotherapy strategies.

In our study, we sought to dissect this complexity by analyzing breast cancer transcriptome and single-cell sequencing data from the TCGA and GEO databases. We identified 54 TSP genes from MSigDB and assessed the activity of the TSP in breast cancer at both the single-cell and bulk transcriptome levels. Using weighted gene co-expression network analysis (WGCNA), we analyzed the module most related to the TSP activity score. Furthermore, we employed a combination of 101 machine learning algorithms to construct a prognostic model capable of predicting patient outcomes, delineating the TME landscape, and estimating the response to ICIs and sensitivity to anti-tumor drugs.

## Materials and methods

### Data collection

**Figure 1:**
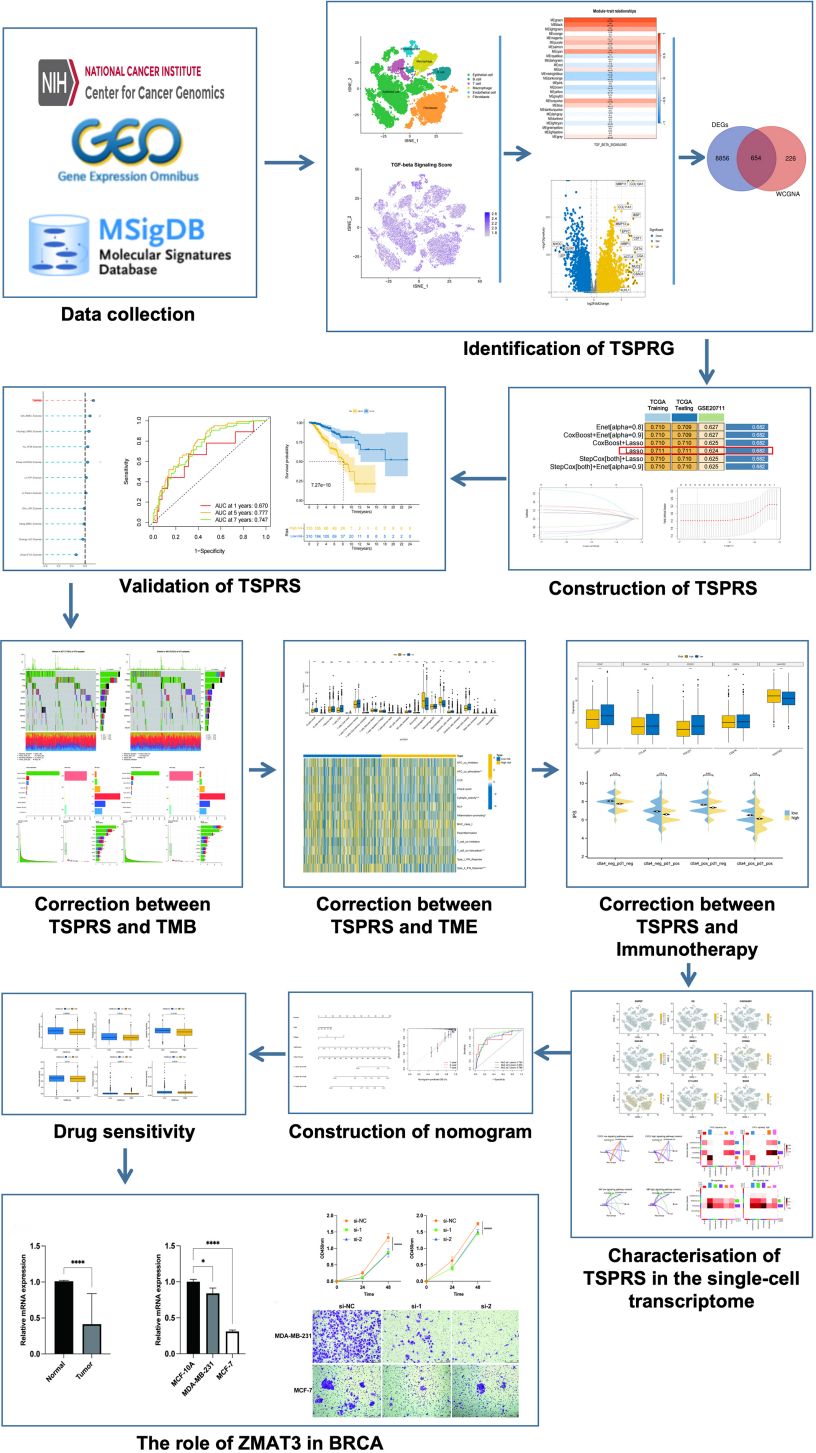
Flowchart for analysis of prognostic signatures associated with the TGF-β signalling pathway. TSPRG, TGF-β signaling pathway-related genes; TSPRS, TGF-β signaling pathway-related signature; TMB, tumor mutation burden.

We sourced a gene expression matrix from 1,081 breast cancer specimens and 99 non-cancerous tissues adjacent to tumors from The Cancer Genome Atlas (TCGA, https://portal.gdc.cancer.gov/) repository. This investigation concentrated on 1,029 breast cancer instances that had comprehensive survival data and a post-diagnosis duration exceeding 30 days. Additionally, we retrieved the GSE20711 cohort, comprising 88 breast cancer cases with exhaustive clinical profiles, from the Gene Expression Omnibus (GEO, https://www.ncbi.nlm.nih.gov/geo/) archive. Furthermore, our study included an analysis of single-cell RNA-sequencing data from five primary breast cancer samples, accessed from the GSE180286 series within the GEO repository. A compilation of 54 genes implicated in the TSP was procured from the Molecular Signatures Database (MSigDB, www.gsea-msigdb.org/gsea/msigdb). **Figure 1** illustrates the flowchart of the data analysis process.

### Processing of single-cell RNAseq data

The "Seurat" package (10) was employed for the analysis of single-cell sequencing data. Initial quality control (QC) involved filtering out cells with mitochondrial gene content exceeding 20% and selecting genes present in a minimum of three cells with expression levels ranging from 200 to 7,500. The next step was to identify a subset of 2,000 highly variable genes for further examination. To correct for batch effects across the various samples, we utilized the “Harmony” algorithm. For the construction of cellular clusters, functions “FindClusters” and “FindNeighbors” within "Seurat" were applied, and the resulting clusters were visualized using the t-distributed Stochastic Neighbor Embedding (t-SNE) technique. Cell type identification and refinement were performed using the “SingleR” tool, guided by known marker genes associated with various cell types.

The activity of specific gene sets within each cell was quantified using the single-sample Gene Set Enrichment Analysis (ssGSEA) method. When comparing differentially expressed genes (DEGs) between two cohorts, the “FindMarkers” utility in "Seurat" was our method of choice. We identified differentially expressed genes with a log fold-change threshold of 0.25 and a minimum percentage of 0.25 while maintaining default settings for other parameters. Additionally, the “CellChat” R package (11) was implemented to explore cellular interactions.

### Weighted gene co-expression network analysis

To conduct the WGCNA analysis on the TCGA-BRCA bulk RNA-seq data, we utilized the “WGCNA” R package (12). Initially, we determined an optimal soft threshold β that adhered to the requirements for constructing a scale-free network. Subsequently, we converted the weighted adjacency matrix into a topological overlap matrix (TOM) and calculated the dissimilarity (dissTOM). For gene clustering and module identification, we employed the dynamic tree-cutting method. Ultimately, we pinpointed the module exhibiting the strongest correlation with the TSP activity scores for further investigation.

### Construction of prognostic signature

We conducted differential analysis between normal and tumor samples in the TCGA bulk RNA-seq data using the ‘DEseq2’ R package, with criteria of |logFC|>0.5 and p.adjust<0.05. Subsequently, we identified the intersection between the DEGs at the bulk RNA-seq level and the genes belonging to the TSP-related module identified through WGCNA. These genes were denoted as TGF-β signaling pathway-related genes (TSPRG). To develop a robust prognostic signature characterized by high predictive accuracy, we followed the subsequent steps:

1. To ensure a balanced distribution of clinical characteristics, we randomly partitioned the TCGA-BRCA dataset into a training set and an internal validation set, maintaining a ratio of 6:4. Additionally, we utilized the GSE20711 dataset as an external validation set.
2. In the TCGA-BRCA training dataset, we conducted univariate Cox regression analysis to identify TSPRG with potential prognostic significance. Subsequently, we employed ten machine learning algorithms, namely CoxBoost, Ridge, Lasso, Random Survival Forest (RSF), Stepwise Cox, Elastic Net (Enet), survival support vector machine (survival-SVM), Generalized Boost Regression Modeling (GBM), Supervised Principal Components (SuperPC), and Partial Least Squares Regression for Cox (plsRcox). To perform variable selection and construct models, we generated 101 combinations of these ten algorithms within the TCGA-BRCA training dataset, employing a tenfold cross-validation framework.
3. We assessed the performance of all constructed models in both the TCGA internal validation set and the GSE20711 dataset. For each model, we computed the concordance index (C-index) across the training, internal validation, and external validation sets. Subsequently, we ranked the models based on their mean C-index to determine their predictive performance. We selected algorithms that demonstrated both robust performance and clinical translational significance. Consequently, we developed a final signature, termed the TGF-β signaling pathway-related signature (TSPRS), which can effectively predict overall survival in BRCA patients.

### Survival analysis and predictive nomogram construction

The TCGA training set, internal validation set, and GSE20711 set were divided into high-risk and low-risk groups based on the median TSPRS risk score. We conducted Kaplan-Meier (KM) curve analysis using the “survminer” R package to assess whether there was a significant difference in overall survival (OS), progression-free survival (PFS), and disease-free survival (DFS) between the high-risk and low-risk groups. Additionally, we performed receiver operating characteristic (ROC) curve analysis using the “timeROC” package to evaluate the sensitivity and specificity of the TSPRS in predicting OS in BRCA patients.

Furthermore, we examined the correlation between the TSPRS and various clinical characteristics, including age, T, M, N, and stage. Univariate and multivariate Cox regression analyses were conducted on the TCGA-BRCA datasets to determine whether the TSPRS served as an independent prognostic factor for predicting survival in BRCA patients.

To enhance the prognostic accuracy and predictive capability of our model, we developed a nomogram that incorporated TSPRS and clinical characteristics to quantify the expected survival of BRCA patients. Finally, we evaluated the precision discrimination and accuracy of the nomogram using ROC curves, the C-index, and calibration curves.

### Tumor mutation burden analyses

We acquired Tumor Mutation Burden (TMB) files containing somatic mutation data from TCGA. We estimated and visualized the differences in TMB levels between the two risk subgroups using the “maftools” R packages. The correlation between risk scores and TMB scores was assessed and depicted using the “limma”, “ggpubr”, “ggplot2”, and “ggExtra” R packages.

KM analysis was employed to examine the survival differences between groups with varying TMB levels as well as between different risk status subgroups. The “Survival” and “survminer” R packages facilitated this analysis.

### Association of TSPRS with tumor microenvironment and response to immunotherapy

To elucidate the relationship between the TSPRS and immune cell infiltration within the BRCA TME, our study utilized computational methodologies including CIBERSORT, ESTIMATE, and ssGSEA. These algorithms were instrumental in quantifying the degree of immune cell infiltration and the activity of immune-related functions.

Furthermore, the Tumor Immune Dysfunction and Exclusion (TIDE) computational framework (accessible at http://tide.dfci.harvard.edu/) was applied to appraise the potential for immune escape mechanisms in patient groups stratified by high and low prognostic risk.

Immune phenotype scores (IPS), retrieved from The Cancer Immunome Atlas (TCIA) database (available at https://tcia.at/home), were used to anticipate the efficacy of immunotherapeutic interventions in TCGA-BRCA patient subsets, delineated by the aforementioned risk stratification.

### TSPRS and drug sensitivity analysis

To facilitate the customization of therapeutic regimens, we employed the “oncoPredict” package (13). This predictive model was applied to estimate the chemosensitivity of patients with BRCA, stratified by varying TSPRS. OncoPredict executes a comparative analysis between the gene expression profiles of patient-derived tissue samples and established cancer cell line repositories, thereby calculating the half-maximal inhibitory concentration (IC50) as a metric of chemotherapeutic potency.

### Cell culture and small-interfering RNA transfection

The human breast cancer cell lines MCF-10A, MDA-MB-231 and MCF-7 were procured from the cell bank of the Chinese Academy of Science. MDA-MB-231 and MCF-7 cells were cultured in high glucose DMEM medium, supplemented with 10% fetal bovine serum (FBS) and 1% Penicillin-Streptomycin Solution (P/S), in an incubator set at 37°C with a 5% CO2 atmosphere. The sequences of small-interfering RNA (siRNA) targeting ZMAT3 were cloned into MDA-MB-231 and MCF-7 cells. Using GP-transfect-Mate (GenePharma, China), the siRNA transfection process was conducted as instructed by the manufacturer. The sequences of the siRNAs are shown in **Table 1**.

**Table 1:** The sequences of the siRNA targeting ZMAT3.

Gene	Sequence
si1-ZMAT3	GGGAATGAGTTTAAGATGA
si2-ZMAT3	GGCTCAGGCTCACTATCAA

### Cell transwell migration testing

After cell transfection was completed, the serum-free cell suspension was spread evenly in the upper chamber of Transwell (Corning, USA), and DMEM medium containing 20% FBS was added to the lower chamber. After 8 hours of incubation, the upper chamber was fixed with 4% paraformaldehyde, and the cells that did not cross the polycarbonate membrane were gently scraped off with a cotton swab. After gentian violet staining and washing with PBS, cells at the bottom of the chambers were photographed in different fields of view using a microscope, and cells were counted using imageJ.

### CCK-8 proliferation assay

The transfected cells were inoculated into 96-well plates at a concentration of 200 μL containing 3000 cells per well, and 3-6 replicate wells were set up in each group. The surrounding circle of wells should not be used as sample wells in principle, and 100 μL of PBS was added to each well. 20 μL of CCK-8 solution was added to each well at 0h, 24h, and 48h, respectively, and the absorbance at 450 nm was measured by enzyme labeling instrument after incubation for 1-4 hours in the incubator.

### mRNA expression analysis

We collected 32 pairs of breast cancer tissue and matched adjacent normal tissue from the Department of Breast Care Surgery, The First Affiliated Hospital, Guangdong Pharmaceutical University.

Total RNA was extracted from clinical samples and cells using TRIzol, after which it was reverse-transcribed into cDNA with a reverse transcription kit (Tsingke,China). The reaction conditions were set at 25°C for 10 minutes, 50°C for 15 minutes, and 85°C for 5 minutes, followed by Real-time Quantitative PCR. The PCR amplification conditions were as follows: pre-denaturation at 95°C for 1 minute, denaturation at 95°C for 30 seconds, and annealing at 60°C for 20 seconds, repeated for 40 cycles. The relative expression of the target gene was calculated using the 2^(-△△Ct) method. The primer sequences are shown in **Table 2**.

**Table 2:** The primer sequences for ZMAT3.

Gene	Primer sequence
ZMAT3	F-ATGCAGCAAATAGCTGTCCTC
	R-GGGACTGGAACAACTGGAGTAG

### Statistical analysis

Data manipulation and statistical analyses were performed utilizing the R statistical computing environment (version 4.1.2). Comparative analyses between distinct groups were executed employing the non-parametric Wilcoxon rank-sum test. To quantify the strength and direction of the associations, Spearman’s rank correlation coefficient was calculated for three sets of relationships: between TMB and risk scores, between immune cell infiltration scores and risk scores, and between immune cell infiltration scores and gene expression profiles, respectively. Statistical significance was established at a p-value threshold of less than 0.05.

## Results

### Differential expression and genetic variation mapping of TGF-β signaling pathway genes in breast cancer

We first performed differential expression analysis of 54 TGF-β signaling pathway genes (TSPG) in breast cancer tissues and adjacent normal tissues. We found that 44 TSPG were differentially expressed (**Supplementary Figures S1A, B**). Protein-protein interaction network analysis constructed on the basis of the String database showed that there was a close association between all TSPG except BCAR3, RRAB31 and SLC20A1 (**Supplementary Figure S1C**). In the subsequent phase of our investigation, we assessed the frequency of somatic mutations within a cohort of 54 TSPG in breast cancer cohorts. A waterfall plot was constructed to illustrate the mutational landscape, which identified CDH1 as the gene with the highest mutation frequency, followed in descending order by ARID4B, TJP1, and APC (**Supplementary Figure S1D**). Furthermore, we scrutinized the prevalence of copy number variations (CNV) within the TSPGs. Our findings indicated a significant amplification in copy numbers predominantly for SMURF2, PPP1CA, and KLF10, while SKI, ID3, and RHOA were the genes most notably subjected to copy number losses (**Supplementary Figure S1E**).

### Characterization of the TSP activity in the single-cell transcriptome

**Figure 2:**
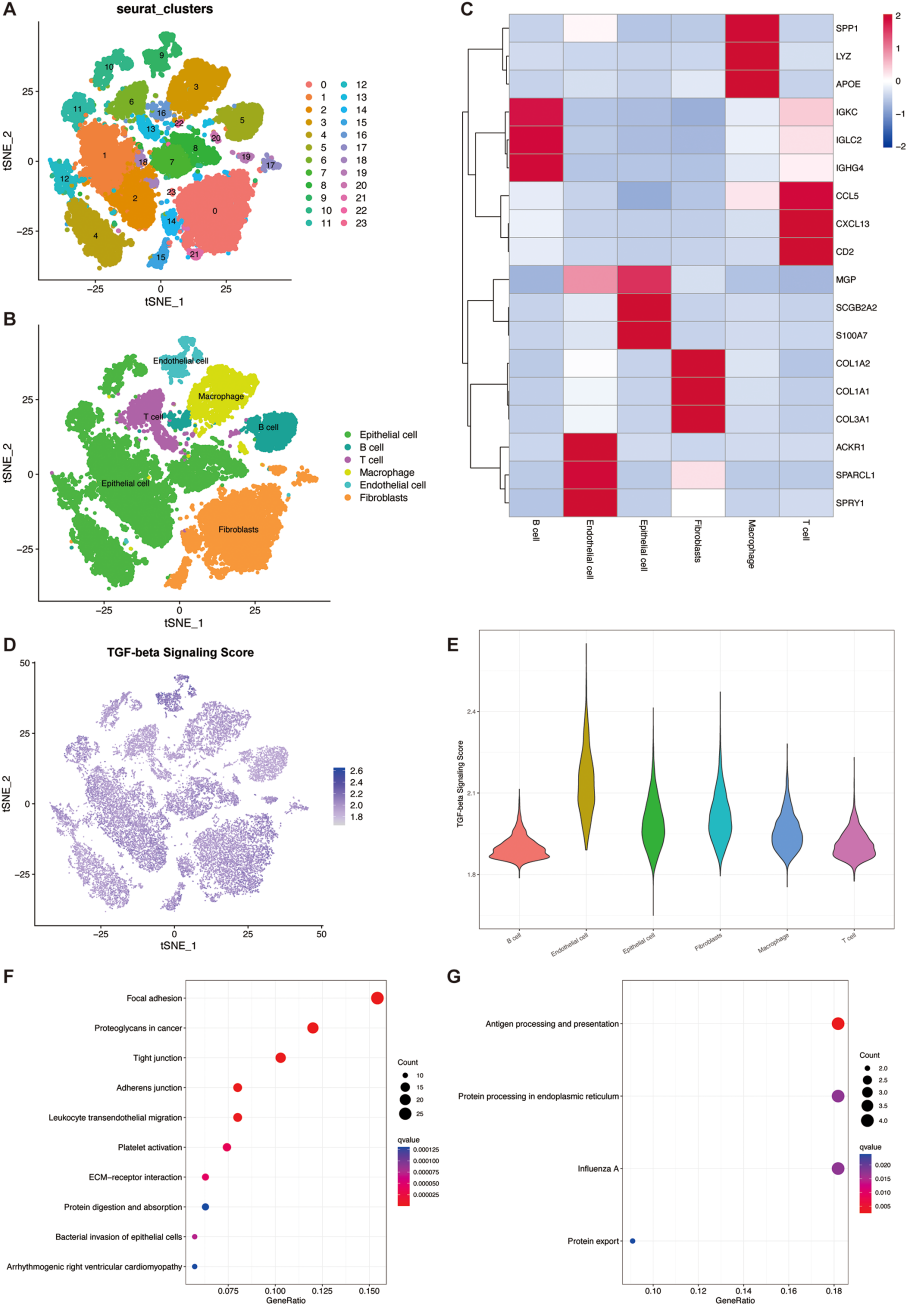
TGF-β pathway activity in the single cell transcriptome. **(A)** t-SNE plot showing the cell clusters. **(B)** t-SNE plot showing the cell types. **(C)** Heatmap showing the top 3 marker genes in each cell types. **(D)** t-SNE plot showing the distribution of TSP scores. **(E)** Violin plot of TGF-β pathway activity scores in each cell type. **(F)** Bubble plots for KEGG enrichment analysis of high TSP score groups. **(G)** Bubble plots for KEGG enrichment analysis of low TSP score groups.

From five primary breast cancer patients, we acquired single-cell RNA-seq data encompassing 25,550 cells. The Harmony algorithm was applied to mitigate batch effects, which facilitated the integration of the quintet of samples. Subsequently, we employed PCA and t-SNE on the most variable 2,000 genes to achieve dimensionality reduction. This stratified the cells into 23 distinct clusters at a resolution parameter of 0.8 (**Figure 2A**). Utilizing marker genes, six clusters were characterized, representative of diverse cellular identities: Epithelial cells, T cells, B cells, macrophages, Endothelial cells, and Fibroblasts (**Figure 2B**). A heatmap was generated to display the three most prominent marker genes within each cellular subset (**Figure 2C**).

In three out of the six cellular phenotypes—namely, Endothelial cells, Fibroblasts, and Epithelial cells—a notably elevated activity of TSP was detected (**Figures 2D, E**). TGF-β signaling plays a crucial role in endothelial cells by regulating angiogenesis, vascular homeostasis, and endothelial function. Through its receptors, TGF-β activates the SMAD pathway, affecting endothelial cell proliferation, migration, and differentiation. In fibroblasts, a primary target of TGF-β, it promotes ECM protein synthesis and fibrosis by inducing collagen and other ECM components, aiding tissue repair and fibrosis. In the TME, fibroblasts, especially cancer-associated fibroblasts (CAFs), use TGF-β signaling to promote tumor progression and metastasis. Additionally, TGF-β regulates epithelial-mesenchymal transition (EMT) in epithelial cells, a process vital for embryonic development, tissue repair, and cancer metastasis, by inducing epithelial cells to acquire mesenchymal traits, enhancing their migratory and invasive capabilities (14–16).

We then identified 322 marker genes in the high TSP activity group and 45 marker genes in the low TSP activity group. Subsequent KEGG enrichment analysis revealed that the marker genes in the high TSP activity group were mainly enriched in focal adhesion, proteoglycans in cancer, and tight junction pathways (**Figure 2F**). The marker genes in the low TSP activity group were mainly enriched in the Antigen processing and presentation pathway (**Figure 2G**).

### Identification of TSP activity-related modular genes

**Figure 3:**
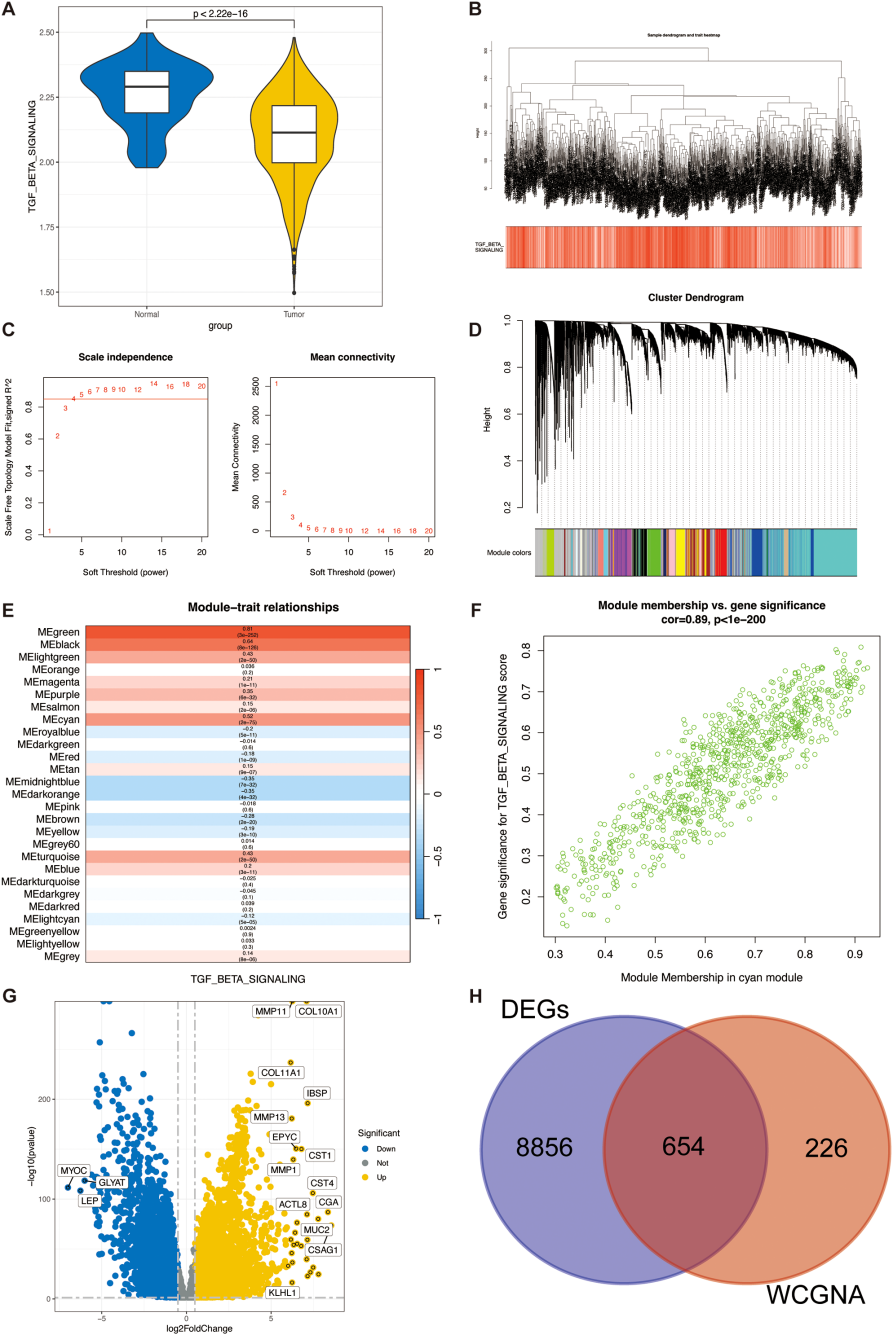
Identification of TSPRG. **(A)** Violin plots of TGF-β pathway activity scores in tumour tissue and adjacent paracancerous normal tissue. **(B)** Dendrogram showing the hierarchical clustering of TCGA-BRCA samples. The bottom heatmap represents the TSPR score of each sample calculated by the ssGSEA algorithm. **(C)** The determination of the optimal soft threshold in WGCNA analysis. **(D)** Cluster dendrogram from the WGCNA analysis. **(E)** Module-trait heatmap showing that the MEgreen module was closely related to the TSPR score feature. **(F)** Scatter plot showing the relationship between gene significance (GS) and module membership (MM) in the green module. **(G)** Volcano plot showing the results of the differential analysis of TCGA-BRCA tumor samples and normal samples. **(H)** Venn plot showing the overlapping genes between the MEgreen module and DEGs in bulk RNA-seq. DEGs, differentially expressed genes; WGCNA, weighted gene co-expression network analysis.

The ssGSEA method is frequently employed to evaluate alterations in biological processes and the activity of pathways within individual specimens. We utilized this algorithm in the present investigation to derive a quantification of TSP activity for each sample within the TCGA-BRCA cohort. Notably, the tumor specimens exhibited a markedly reduced TSP activity in comparison to the adjacent non-tumor tissues (as depicted in **Figure 3A**). This derived score was then incorporated as a phenotypic variable for downstream WGCNA. The WGCNA approach was subsequently applied to the TCGA-BRCA data to pinpoint gene modules that show significant correlations with TSP activity. After removing outlier samples, we constructed co-expression networks (**Figure 3B**). The optimal soft threshold of power=4 was selected to ensure a scale-free topological network (**Figure 3C**). A total of 27 modules were obtained by setting the minimum module gene count to 50 and MEDissThres to 0.15 (**Figure 3D**). Our findings indicated that the MEgreen module was strongly correlated with the TSP activity score in bulk RNA-seq (**Figure 3E**). Moreover, the scatter plot of gene significance (GS) versus module membership (MM) for the blue module displayed a significant correlation (**Figure 3F**), suggesting that genes within the green module may have a functional significance associated with the TSP.

The volcano plot (**Figure 3G**) illustrates the DEGs between breast tumor and adjacent normal tissues in the TCGA-BRCA bulk RNA-seq (|logFC|>0.5 and p.adjust<0.05). We intersected the 880 genes in the green module with the DEGs from the bulk RNA-seq, finally identifying a total of 654 genes (**Figure 3H**), named TSPRG. GO enrichment analysis of TSPRG showed a significant enrichment in biological processes (BP), including extracellular matrix organization and extracellular structure organization, as well as in cellular components (CC) such as collagen-containing extracellular matrix, and in molecular functions (MF) including extracellular matrix structural constituent (**Supplementary Figure S2A**). Kyoto Encyclopedia of Genes and Genomes (KEGG) enrichment analysis of TSPRG showed a significant enrichment in Focal adhesion, Protein digestion and absorption, Proteoglycans in cancer, and ECM−receptor interaction (**Supplementary Figure S2B**).

### Development of a prognosis signature through comprehensive machine learning integration

**Figure 4:**
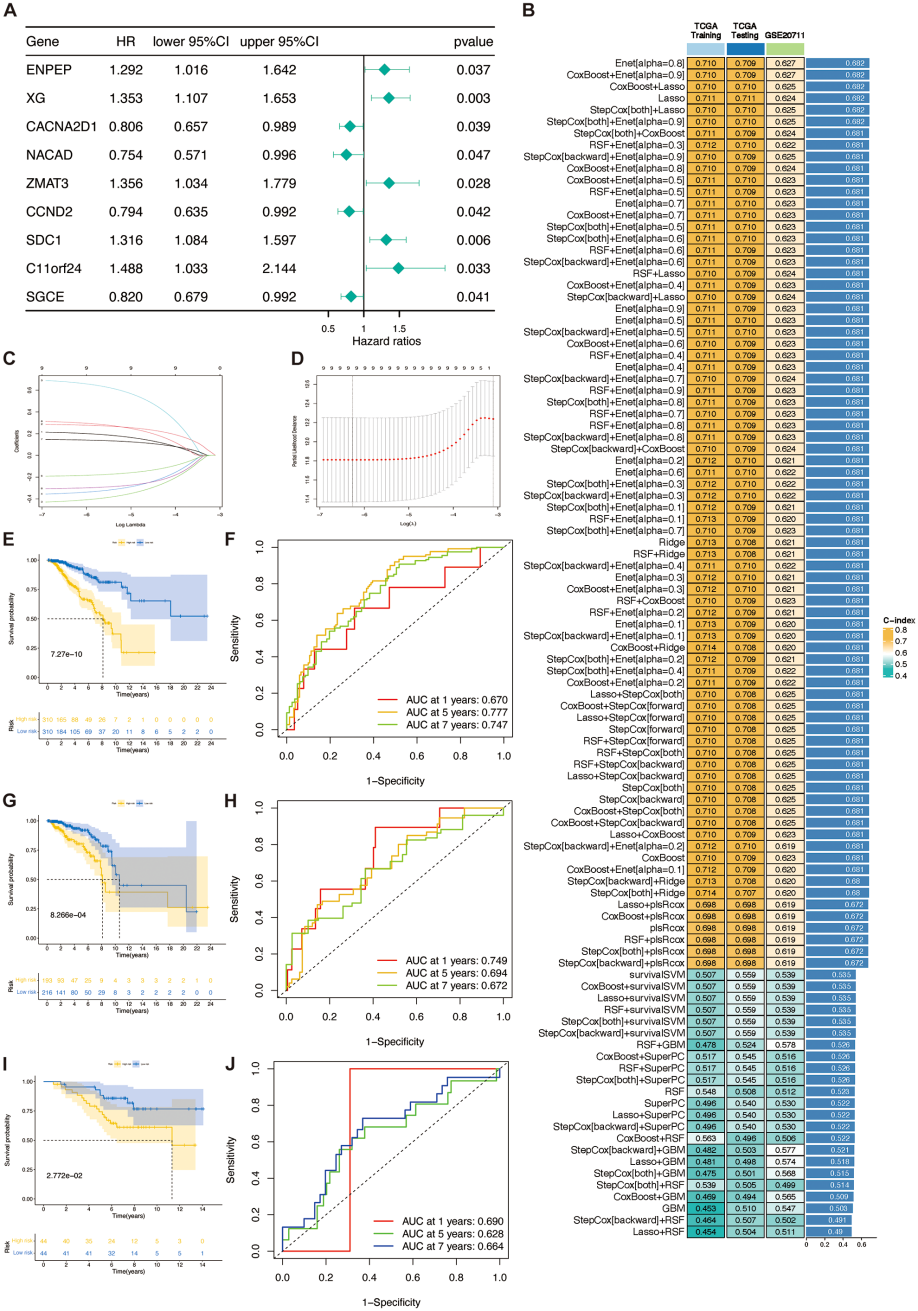
TSPRS was developed and validated using machine learning. **(A)** Forest plots showing the results of univariate Cox regression analysis. **(B)** A total of 101 kinds of prediction models via a tenfold cross-validation framework and further calculating the C-index of each model across all validation datasets. **(C, D)** Visualization of the LASSO regression in the TCGA training set. The optimal λ was obtained when the partial likelihood deviation reached the minimum value. **(E, G, I)** Kaplan-Meier curves of OS according to TSPRS in the TCGA training set, TCGA internal validation set, and GSE20711. **(F, H, J)** ROC curves showing the specificity and sensitivity of TSPRS in predicting 1, 5, and 7-year OS in the TCGA training set, TCGA internal validation set, and GSE20711.

To develop a unified signature associated with the TSP, we employed an ensemble of 101 machine learning algorithms to evaluate the nine genes identified as prognostic by univariate Cox regression analysis (**Figure 4A**). Within the TCGA training cohort, we constructed 101 predictive models using a ten-fold cross-validation scheme and calculated the C-index for each combination of training and validation groups (**Figure 4B**).

Out of the 101 models, we calculated the C-index of each machine learning algorithm combination in the three datasets and ranked each algorithm combination according to its average C-index. The results show that the six algorithm combinations, Enet [alpha=0.8], CoxBoost+Enet [alpha=0.9], CoxBoost+Lasso, Lasso, StepCox[both]+Lasso, and StepCo[both]+Enet [alpha=0.9], exhibit good prediction ability. However, among these six algorithm combinations, only the Lasso algorithm showed the best prediction ability in the training set and the internal validation set. The Lasso algorithm achieved an area under curve (AUC) of 0.711 on both the TCGA training and test sets, and 0.624 on the GSE20711 validation set. These results show that Lasso’s performance is on par with other models like Enet and CoxBoost, and it remains consistent across datasets. By reducing some coefficients to zero for feature selection, the Lasso model simplifies and enhances interpretability, focusing on identifying key genes in the TSP. These benefits led us to select the Lasso model for constructing TSPRS.

Using a tenfold cross-validation framework, we identified the optimal λ value of 0.001900647 in the LASSO analysis by minimising the partial likelihood deviation (**Figures 4C, D**). A prognostic signature was developed employing the nine genes that retained non-zero coefficients subsequent to the LASSO regression analysis (**Table 3**). For each patient, a risk score was computed, facilitating the stratification of patients into high-risk or low-risk categories predicated on the median value of the calculated risk scores. Kaplan-Meier curves show that SDC1, NACAD, ZMAT3, CCND2, XG and SGCE are associated with prognosis in breast cancer patients (**Supplementary Figures S3A–I**). It was observed that there was a proportional escalation in mortality concomitant with an increase in the risk score (**Supplementary Figures S4A–L**). Furthermore, comparative analyses of OS rates within the training cohort, the internal validation cohort, and the external GSE20711 dataset revealed that patients categorized within the high-risk bracket had significantly inferior OS outcomes compared to their low-risk group (**Figures 4E, G, I**). In a similar vein, assessments of PFS and DFS demonstrated a markedly improved prognosis for patients in the low-risk group relative to those in the high-risk group (**Supplementary Figures S4O, P**).

**Table 3:** The nine genes that constitute TSPRS.

Gene Symbol	Full Name	Function	Known Biological Role in Breast Cancer	References
ENPEP	Glutamyl Aminopeptidase	Involved in blood pressure regulation.	May be related to tumor microenvironment and angiogenesis.	(41)
XG	Xg Glycoprotein	Blood group antigen	Specific role in breast cancer is unclear.	
CACNA2D1	Calcium Voltage-Gated Channel Auxiliary Subunit Alpha2delta 1	Regulates calcium channel activity.	Increasing the infiltration of immune cells and up-regulating the expression of immune checkpoints.	(42)
NACAD	NAC Alpha Domain Containing	Involved in protein transport	Specific role in breast cancer is unclear.	(43)
ZMAT3	Zinc Finger Matrin-Type 3	Involved in p53 signaling pathway	May play a role in tumor suppression and cell cycle regulation.	(40)
CCND2	Cyclin D2	Regulates cell cycle	Promotes cell proliferation, potentially related to breast cancer progression.	(44)
SDC1	Syndecan 1	Participates in cell proliferation, cell migration, and cell-matrix interactions through its receptor for extracellular matrix proteins.	Interacts with various ligands and receptors involved in tumor progression, affecting cancer stem cell function, cell proliferation, etc.	(45, 46)
C11orf24	Chromosome 11 Open Reading Frame 24	Involved in cell cycle progression.	Specific role in breast cancer is unclear.	(47)
SGCE	Sarcoglycan Epsilon	A single pass transmembrane protein forming part of the dystrophin-associated glycoprotein complex.	Specific role in breast cancer is unclear.	(48)

### Validation of TSPRS

The analysis of the ROC curve indicated that for the training cohort, the AUC for the TSPRS was 0.670, 0.777, and 0.747 at 1, 5, and 7 years, respectively. For the internal validation cohort, these values were 0.749, 0.694, and 0.672, and for the GSE20711 dataset, the AUCs were 0.690, 0.628, and 0.664 (**Figures 4F, H, J**). These metrics underscore the superior prognostic efficacy of the TSPRS.

**Figure 5:**
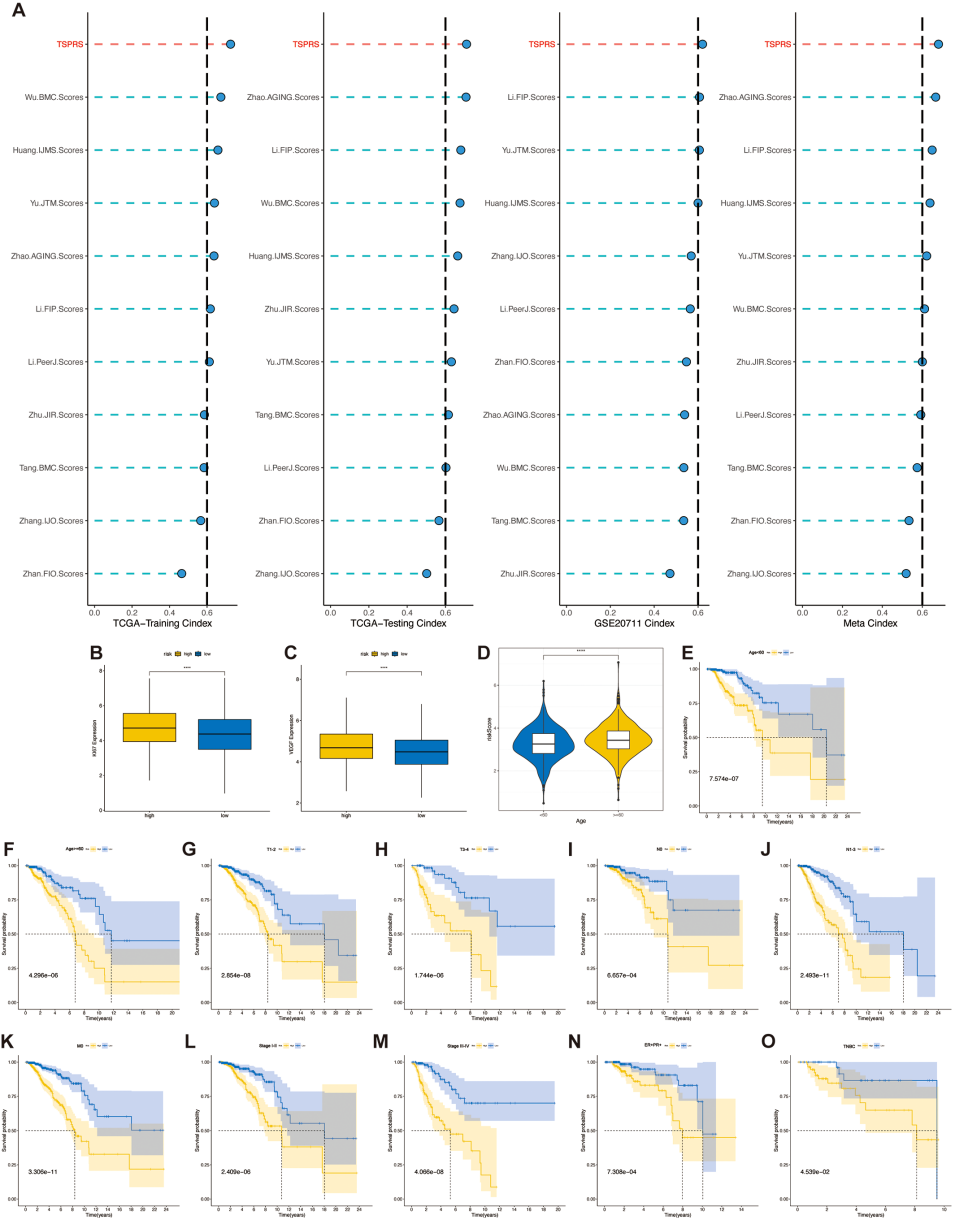
Clinical relevance of TSPRS. **(A)** C-index comparison of TSPRS with ten published prognostic signatures for breast cancer patients. **(B)** Differences in KI-67 expression levels between high and low risk groups. **(C)** Differences in VEGF expression levels between high and low risk groups. **(D)** Differences in risk scores between patients younger than 60 years and those older than or equal to 60 years. **(E-O)** Kaplan-Meier curves showing the stable performance of TSPRS in the subgroups of BRCA patients, including age, T, N, M, stage, ER+/PR+ and TNBC. ****, P<0.0001.

The comparative analysis of prognostic performance was conducted between TSPRS and ten established prognostic signatures, as devised by Wu et al., Zhan et al., Huang et al., Li et al. (first instance), Yu et al., Tang et al., Li et al. (second instance), Zhang et al., Zhu et al., and Zhao et al. (17–26), with respect to patient outcomes in BRCA. Our analysis revealed that TSPRS surpasses the aforementioned prognostic models, evidenced by a significantly elevated C-index (**Figure 5A**). These findings reinforce the remarkable prognostic precision of TSPRS in the context of anticipating clinical outcomes for BRCA patients.

Then we observed the expression of the angiogenic marker VEGF and the cell proliferation indicator KI67 in the high- and low-risk groups, and the results showed that there were higher levels of VEGF and KI67 expression in the high-risk group, suggesting that the tumors of the patients in the high-risk group had higher levels of angiogenic and proliferative capacity than those in the low-risk group (**Figures 5B, C**).

Given the routine utilization of clinical characteristics to prognosticate outcomes for patients with BRCA, we conducted an assessment of the association between the TSPRS and a spectrum of clinical parameters. In the TCGA-BRCA dataset, we observed significantly higher risk scores in patients with age greater than or equal to 60 than in patients with age less than 60 (**Figure 5D**), but did not observe differences in risk scores in stage, T, N, or M (**Supplementary Figures S5A–E**). We also found that TSPRS showed strong prognostic power in ER+/PR+ patients, TNBC patients, and subgroups with different clinicopathologic features (**Figures 5E–O**). However, this good predictive ability was not observed in patients with M1-staged and HER2+ breast cancer, probably due to the small sample size (**Supplementary Figures S5F, G**).

### Association of TSPRS with mutation landscapes

TMB is generally defined as the number of non-synonymous mutations per megabase pair (Mb) of somatic cells in a given genomic region. Tumor mutational load is a quantitative biomarker that reflects the total number of mutations carried by tumor cells, indirectly reflecting the ability and degree of neoantigen production by tumors, and has been shown to predict the efficacy of immunotherapy for a variety of tumors (27, 28). Tumor cells with a high TMB have a higher level of neoantigens, and studies have shown that patients with a high TMB are more likely to benefit from ICIs therapy (29).

**Figure 6:**
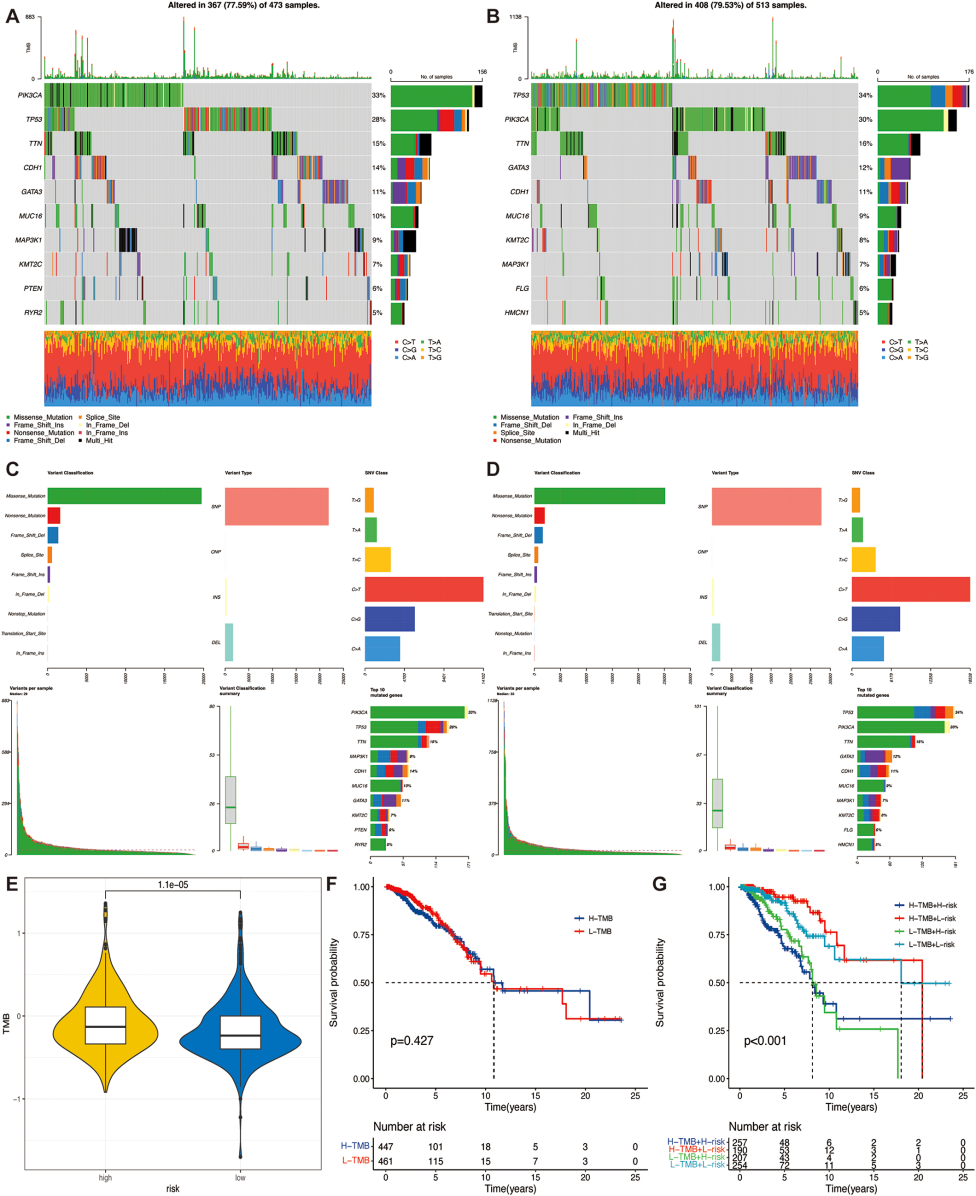
Correlation of TSPRS with TMB. **(A)** Waterfall plot of the top ten genes’ TMB status in the low-risk groups, and **(B)** the high-risk group. **(C)** Summary of the maf files of the low-risk groups, and **(D)** the high-risk group. **(E)** Differences in TMB between high and low risk groups. **(F)** Kaplan-Meier curve of different TMB levels. **(G)** Kaplan-Meier curves of different TMB and risk levels. TMB, tumor mutation burden.

The ten genes with the highest mutation rates differed significantly between the two groups (**Figures 6A–D**). TMB was significantly higher in the high-risk group than in patients in the low-risk group (**Figure 6E**), and there was a significant positive correlation between the risk score and TMB (**Supplementary Figure S4S**). Suggesting that patients in the high-risk group may benefit from ICIs treatment. To understand whether the risk profile or TMB was a better predictor of survival, we divided the samples into high and low mutation subgroups based on median TMB. KM survival curves showed no statistically significant difference between the survival of patients in these two groups (**Figure 6F**). However, in the combined analysis of TMB and TSPRS, both low TMB in the high-risk group and high TMB in the high-risk group possessed shorter survival times, suggesting a worse prognosis (**Figure 6G**). This suggests the stronger predictive ability of TSPRS.

### Association of TSPRS with the breast cancer microenvironment

To evaluate the status of immune cell infiltration within breast cancer specimen, we utilized the Estimation of Stromal and Immune cells in Malignant Tumor tissues using Expression data (ESTIMATE) algorithm. This computational approach facilitated the quantification of the immune score, stromal score, ESTIMATE score, and an estimation of tumor purity across different TSPRS risk stratifications. Contrary to expectations, a comparative analysis between high-risk and low-risk cohorts did not yield statistically significant differences for these four scores (**Supplementary Figures S5I–L**).

**Figure 7:**
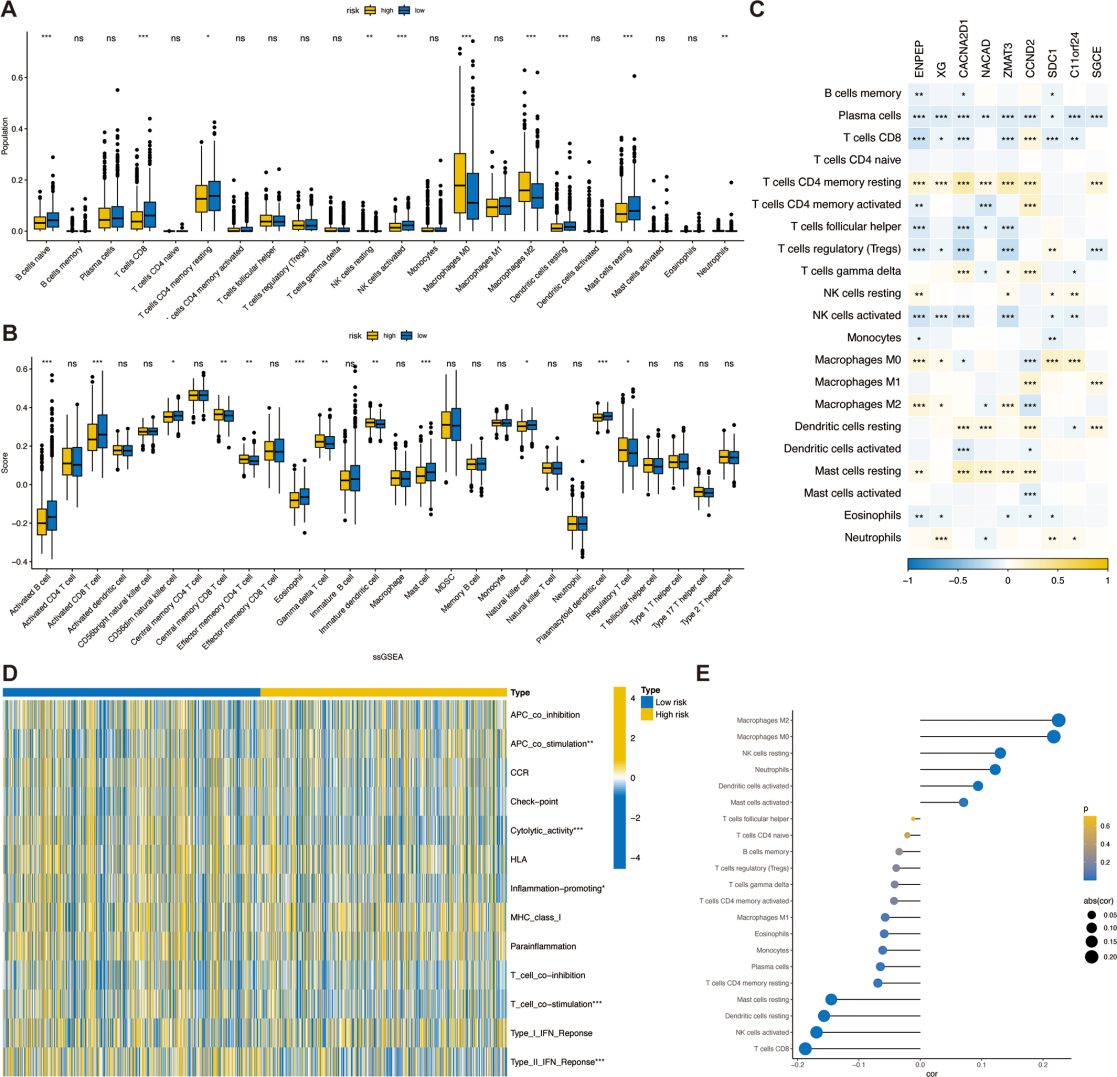
Association of TSPRS with the TME in breast cancer.The abundance of each TME-infiltrated cell type between high- and low risk groups, quantified by the **(A)** CIBERSORT algorithm and the **(B)** ssGSEA algorithm. **(C)** The correlation between TME infiltrating cells and TSPRS genes. **(D)** Heatmap of differences in activity of immune-related pathways between high- and low-risk groups. **(E)** Correlation analysis between TME infiltrated cells and TSPRS scores. *, P<0.05; **, P<0.01; ***, P<0.001; ns, P≥0.05. TSPRS, TGF-β signaling pathway-related signature; TME, tumor microenvironment.

To delve into the disparities in the infiltration of specific immune cell types between the high- and low-risk groups, we quantitatively assessed the prevalence of various infiltrating immune cells within each sample by employing the CIBERSORT algorithm. This analytical tool allows for a refined characterization of the immune cell composition by deconvolving gene expression profiles from bulk tumor transcriptomic data. We found lower levels of CD8+ T cells, NK cells activated and higher levels of M2 macrophages in the high-risk group compared to the low-risk group. This suggests a higher level of immunosuppression in the high-risk group (**Figure 7A**). Concordant outcomes were procured through the application of the ssGSEA algorithm, serving as a validation method (**Figure 7B**). Moreover, within the TSPRS, we identified nine genes that exhibited a strong correlation with the presence of tumor-infiltrating immune cells. Notably, *ENPEP, XG, CACNA2D1, ZMAT3*, and *SDC1* demonstrated a significant inverse correlation with activated CD8+ T cells and NK cells. Additionally, *ENPEP, XG*, and *ZMAT3* showed a significant positive correlation with M2 macrophages (**Figure 7C**).

CD8+ cytotoxic T lymphocytes (CTLs) are the preferred immune cells for targeting cancer. However, during cancer progression, immune tolerance and suppression within the TME lead to CTL dysfunction and exhaustion, promoting adaptive immune resistance. M2 macrophages and regulatory T cells (Tregs) form immune barriers against CD8+ T cell-mediated antitumor immune responses. In the breast cancer microenvironment, TGF-β can induce macrophages to transform into M2 macrophages, which possess immunosuppressive and tumor-promoting characteristics. These cells secrete various immunosuppressive factors, including TGF-β. TGF-β inhibits CXCR3 expression in CD8+ T cells (30), thereby limiting their infiltration into tumors. This finding is consistent with our research results. Given the role of TGF-β signaling in the immunosuppressive TME, combining TGF-β signaling inhibitors with ICIs can enhance antitumor immune responses.

Additionally, using the ssGSEA algorithm, we obtained immune-related pathway scores. The high-risk group demonstrated significantly stronger activity in APC co-stimulation. The low-risk group demonstrated significantly stronger activity in cytolytic activity, inflammation promoting, T cell co-stimulation, and type II IFN response pathways (**Figure 7D**).

Next, we investigated the correlation between TSPRS and immune cell infiltration by Spearman’s correlation analysis, and in agreement with previous results, the risk score was positively correlated with M2 macrophages, and negatively correlated with NK cells activated and CD8+ T cells, suggesting that the risk score predicts the immune status in breast cancer tissues (**Figure 7E**).

### The relationship between the TSPRS and immunotherapy response

**Figure 8:**
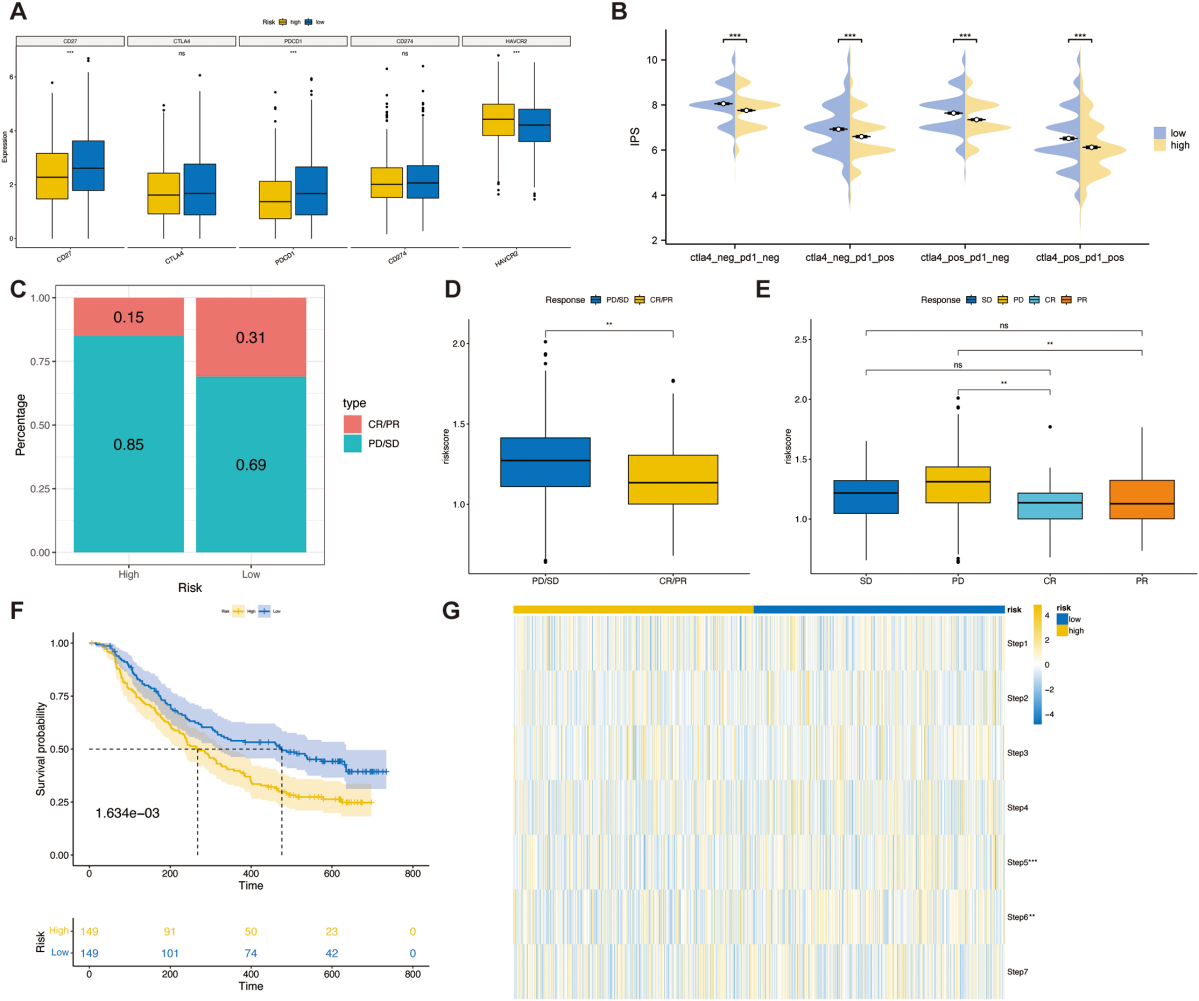
The relationship between TSPRS and immunotherapy response. **(A)** The expression of immune checkpoints in high-risk and low-risk groups. **(B)** The IPS score between high-risk and low-risk groups. **(C)** The proportion of CR/PR or SD/PD patients receiving immunotherapy in the high- and low-risk groups of the IMvigor210 cohort. **(D)** A boxplot showing the difference in risk score between patients with CR/PR and those with SD/PD in the IMvigor210 cohort. **(E)** A boxplot showing the variation in risk score between patients with CR, PR, SD and PD in the IMvigor210 cohort. **(F)** KM survival curves for high and low risk groups in the IMvigor210 cohort. **(G)** Heatmap showing the diference in the seven-step anti-cancer immunity cycle activity between high- and low-risk groups. **, P<0.01; ***, P<0.001; ns, P≥0.05. CR, complete response; PD, progressive disease; PR, partial response; SD, stable disease.

Prior studies have linked higher immune checkpoint levels to better responses to ICIs (25). We compared immune checkpoint expression in TSPRS risk subgroups. The results indicated that in the high-risk group, CD27 and PDCD1 expression levels were lower, while HAVCR2 expression levels were higher, compared to those in the low-risk group (**Figure 8A**). Further analysis of IPS scores from the TCIA database showed higher scores correlate with improved ICI response, including treatments with PD-1 and CTLA4 inhibitors across four categories: (1) ips_ctla4_pos_pd1_pos, (2) ips_ctla4_pos_pd1_neg, (3) ips_ctla4_neg_pd1_pos, and (4) ips_ctla4_neg_pd1_neg. Our findings reveal that the low-risk group had significantly higher IPS scores and better responses to all four ICIs treatment regimens compared to the high-risk group (**Figure 8B**).

To validate TSPRS’s prognostic value in predicting immune therapy outcomes, we analyzed the IMvigor210 cohort treated with atezolizumab. Using TSPRS, we assigned risk scores and categorized patients into high-risk and low-risk groups. The low-risk group showed a higher rate of complete or partial responses (CR/PR), whereas the high-risk group mainly had progressive or stable disease (PD/SD, **Figure 8C**). Risk scores were significantly lower in patients with PD/SD compared to those with PD/SD (**Figure 8D**). Specifically, patients with progressive disease (PD) had notably higher risk scores than those achieving responses (**Figure 8E**). In the IMvigor210 dataset, shorter survival times were associated with the high-risk group (**Figure 8F**). These results support TSPRS’s ability to forecast outcomes of immune-based treatments, suggesting better therapeutic outcomes for low-risk patients.

Finally, we evaluated the activity of the anticancer immune cycle in high and low risk groups by the Tracking Tumor Immunophenotype (TIP) database in order to gain a comprehensive understanding of the anticancer role of immune cells and to guide immunotherapy (31). The seven steps are release of cancer cell antigens (Step 1), cancer antigen presentation (Step 2), priming and activation (Step 3), trafficking of immune cells to tumors (Step 4), infiltration of immune cells into tumors (Step 5), recognition of cancer cells by T cells (Step 6) and killing of cancer cells (Step 7). The results showed that the activities of Steps 5 and 6 of the anti-cancer immune cycle were significantly higher in the low-risk group than in the high-risk group (**Figure 8G**).

### The correlation of the TSPRS with single-cell characteristics

**Figure 9:**
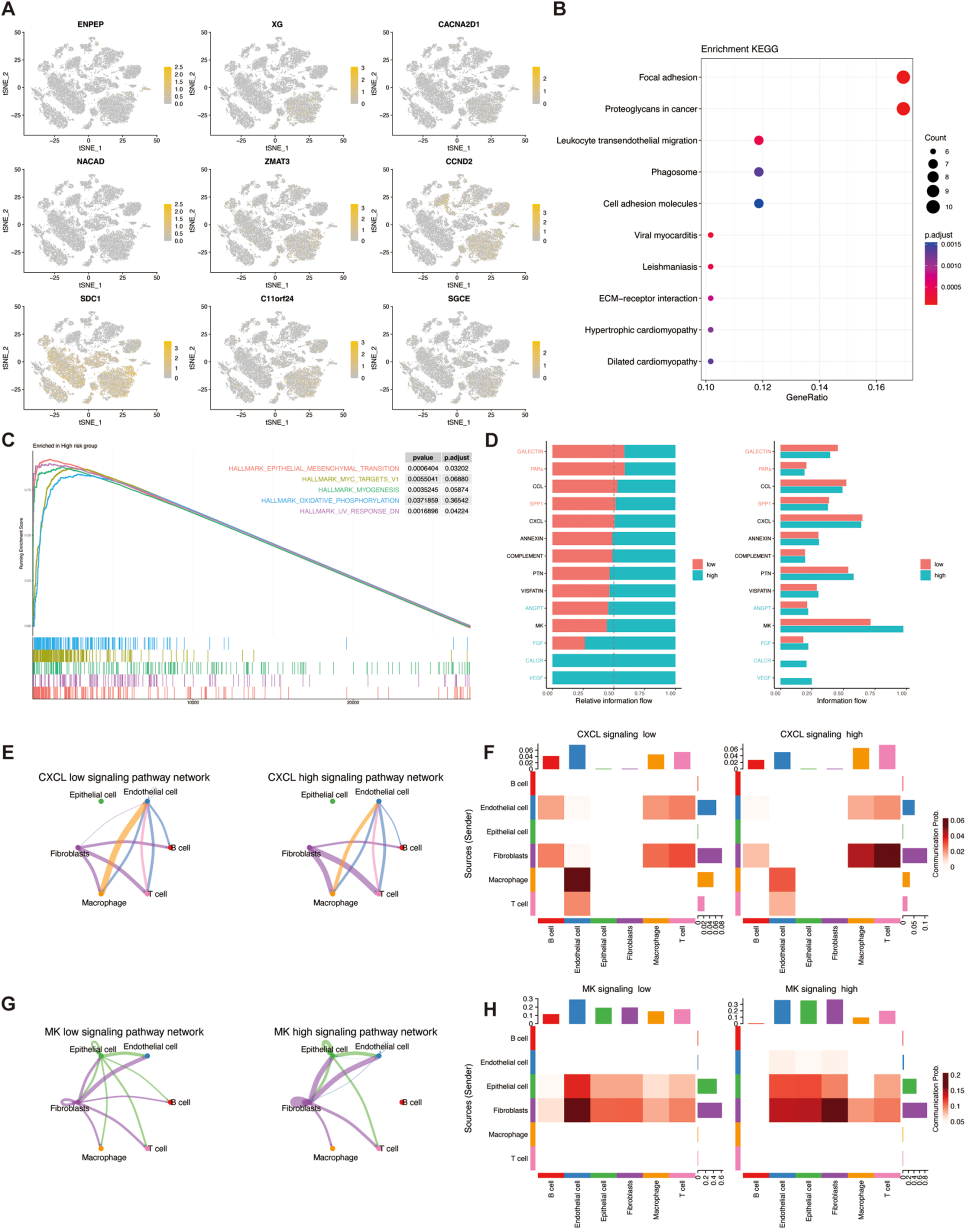
The correlation of TSPRS with single-cell characteristics. **(A)** The expression of ENPEP,XG,NACAD,CACNA2D1,ZMAT3,CCND2,SDC1,C11orf24 and SGCE in different cell types by single-cell RNA-seq analysis. **(B)** KEGG analysis of DEGs between the high-risk and low-risk cells. **(C)** GESA enrichment analysis of the high-risk groups. **(D)** Comparative analysis of the intensity of cellular signaling pathways in high- and low-risk groups. **(E)** CXCL pathway network loops in high and low risk groups. **(F)** Heatmap of the role of different cell types in the CXCL pathway network in high and low risk groups. **(G)** MK pathway network loops in high and low risk groups. **(H)** Heatmap of the role of different cell types in the MK pathway network in high and low risk groups. KEGG, Kyoto Encyclopedia of Genes and Genomes; GSEA, Gene Set Enrichment Analysis.

To elucidate the influence of TSPRS within the TME at the level of single-cell transcriptomics, we conducted an examination of the expression profiles of a cohort of genes, namely *ENPEP, XG, CACNA2D1, NACAD, ZMAT3, CCND2, SDC1, C11orf24*, and *SGCE*, across diverse cellular phenotypes. Our findings delineated that the aforementioned genes predominantly manifested expression within fibroblasts, with CCND2 additionally exhibiting pronounced expression in T cells (**Figure 9A**).

KEGG enrichment analysis showed that the differential genes in the high and low risk groups were mainly enriched in the pathways of Focal adhesion, Proteoglycans in cancer, and Leukocyte transendothelial migration (**Figure 9B**). Gene Set Enrichment Analysis (GSEA) enrichment analysis showed that the high-risk group was mainly involved in pathways such as EPITHELIAL_MESENCHYMAL_TRANSITION (**Figure 9C**).

In addition, we found that cells within the high- and low-risk groups had different communication patterns (**Figure 9D**). In the CXCL pathway, the high-risk group was dominated by macrophage-endothelial cell communication, whereas the low-risk group was dominated by fibroblast-T cell communication (**Figures 9E, F**). In the MK pathway, the high-risk group was dominated by fibroblast-fibroblast communication, whereas the low-risk group was dominated by fibroblast-endothelial cell communication (**Figures 9G, H**).

### Establishment and validation of a nomogram

**Figure 10:**
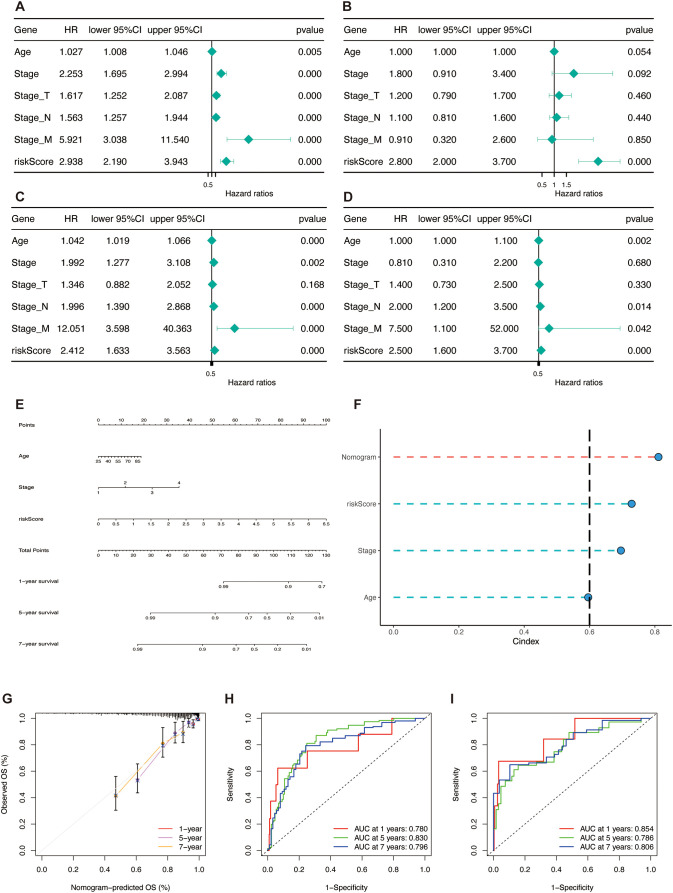
Construction and validation of the nomogram. **(A, B)** Univariate and multivariate Cox regression analyses of clinical pathology variables and risk scores with OS in the TCGA training set and **(C, D)** the TCGA test set. **(E)** Construction of the nomogram based on TSPRS and clinical characteristics, including age and stage. **(F)** C-index comparison of the nomogram, TSPRS, stage and age. **(G)** Calibration curve of the nomogram for 1, 5 and 7-year OS. **(H)** ROC curves showing the predictive performance of the nomogram in 1, 5, and 7-year OS in the TCGA training set and **(I)** the TCGA testing set.

To evaluate the independence of TSPRS as a prognostic indicator for BRCA, a series of Cox proportional hazards regression analyses—both univariate and multivariate—were performed on overall survival (OS) metrics within the TCGA-BRCA (**Figures 10A–D**). The results demonstrated that TSPRS constituted a considerable hazard element for OS based on the single-variable analysis. Further, TSPRS preserved its status as an autonomous prognostic indicator in the multiple-variable analysis, suggesting its strong predictive capacity for BRCA patient outcomes.

To enhance the clinical utility of the TSPRS, stepwise regression was employed to examine the interplay between clinical attributes and TSPRS. This analysis facilitated the integration of age, disease stage, and TSPRS into a predictive nomogram (**Figure 10E**). The C-index affirmed the nomogram’s consistent and formidable predictive performance, surpassing that of other clinical parameters (**Figure 10F**). Calibration plots corroborated the congruence between the nomogram’s forecasts and the actual clinical outcomes (**Figure 10G**). Within the training cohort, the AUC values for the nomogram were impressive, registering at 0.780, 0.830, and 0.796 for the 1-, 5-, and 7-year marks, respectively (**Figure 10H**). The internal validation set yielded AUC values of 0.854, 0.786, and 0.806 at the corresponding 1-, 5-, and 7-year intervals, underscoring the nomogram’s high predictive precision (**Figure 10I**). Collectively, these results endorse the TSPRS-informed nomogram as a robust and precise instrument for the tailored prognostication of BRCA patients.

### Analysis of the correlation between the TSPRS and drug sensitivity

**Figure 11:**
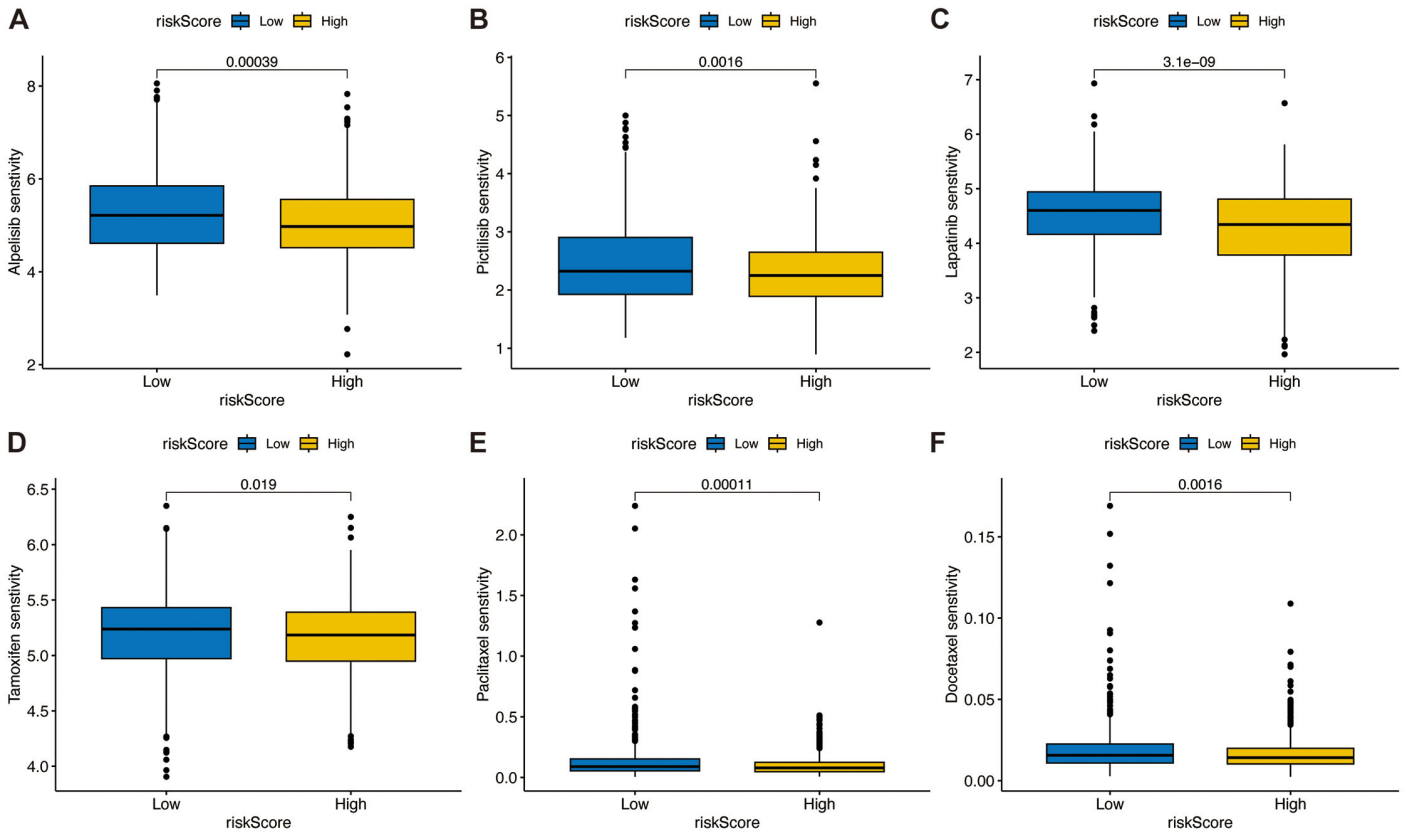
Association between the TSPRS and drug sensitivity. The box plots show the half-maximal inhibitory concentration of PIK3CA kinase inhibitors **(A)** Alpelisib and **(B)** Pictilisib, the tyrosine kinase inhibitor **(C)** Lapatinib, the estrogen receptor modulator **(D)** Tamoxifen, and conventional chemotherapy agents **(E)** Paclitaxel and **(F)** Docetaxel in high and low risk groups.

We analyzed the correlation between TSPRS and drug sensitivity using the R package ‘oncoPredict’, which showed that the IC50 of the high-risk group for the PIK3CA kinase inhibitors Alpelisib and Pictilisib (**Figures 11A, B**), the tyrosine kinase inhibitor Lapatinib (**Figure 11C**), the estrogen receptor modulator Tamoxifen (**Figure 11D**), and the conventional chemotherapeutic agents Paclitaxel and Docetaxel were lower than those of the low-risk group (**Figures 11E, F**).

### ZMAT3 is associated with breast cancer growth and invasion

**Figure 12:**
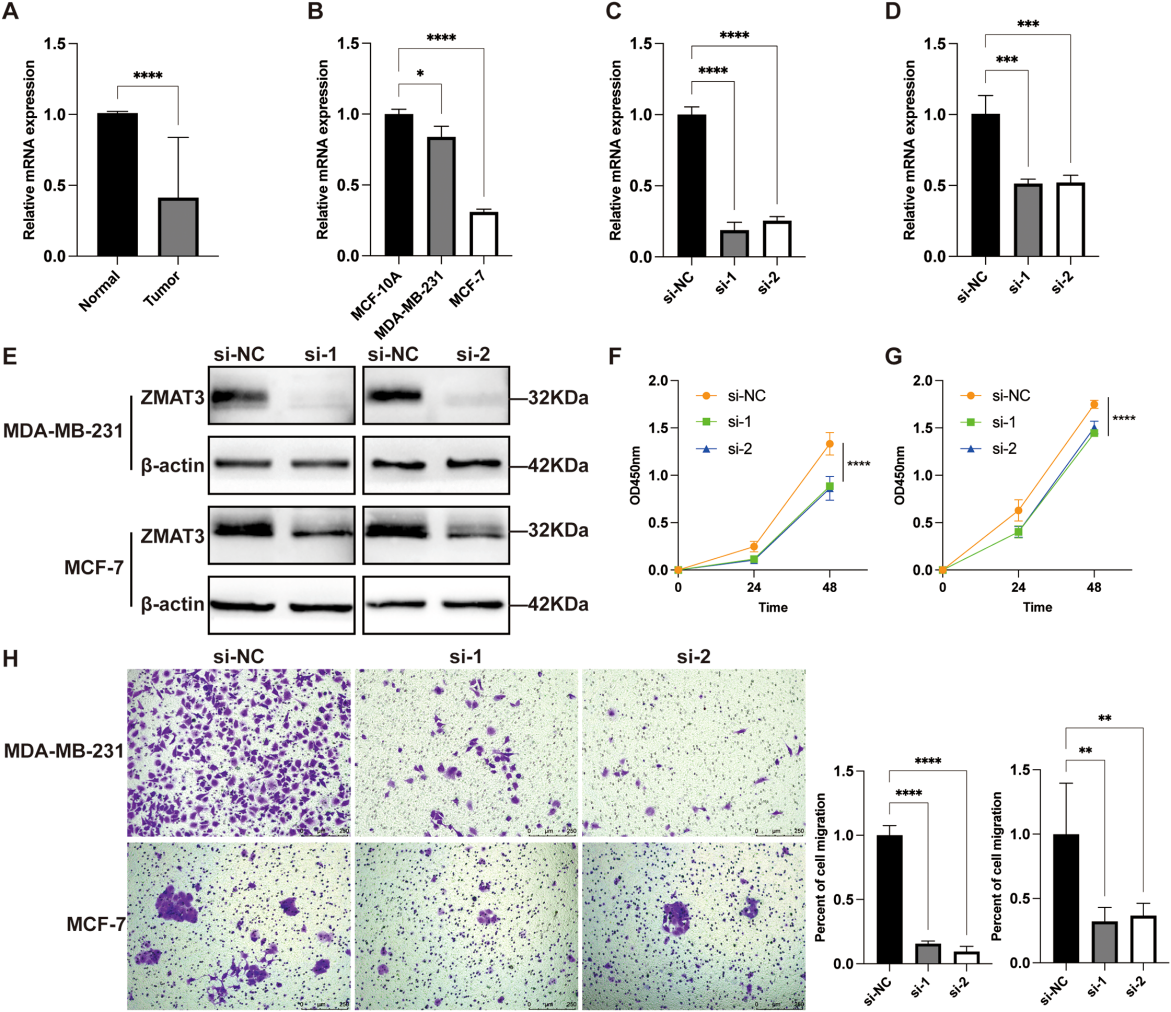
The role of ZMAT3 in breast cancer. **(A)** Expression levels of ZMAT3 in tumor tissues and normal tissues in 32 matched pairs of clinical samples. **(B)** Expression levels of ZMAT3 in MCF-10A, MDA-MB-231 and MCF-7 cells. **(C)** Validation of ZMAT3 knockdown at the mRNA level in MDA-MB-231 cells. **(D)** ZMAT3 knockdown was verified at the mRNA level in MCF-7 cells. **(E)** ZMAT3 knockdown was verified at the protein level in MDA-MB-231 and MCF-7 cells. **(F)** CCK-8 assay to detect changes in proliferative capacity of MDA-MB-231 cells after ZMAT3 knockdown. **(G)** CCK-8 assay to detect changes in proliferative capacity of MCF-7 cells after knockdown of ZMAT3. **(H)** Migration assay to detect changes in invasion ability of MDA-MB-231 and MCF-7 cells after knockdown of ZMAT3. *, P<0.05; **, P<0.01; ***, P<0.001; ****, P<0.0001.

**Figure 13:**
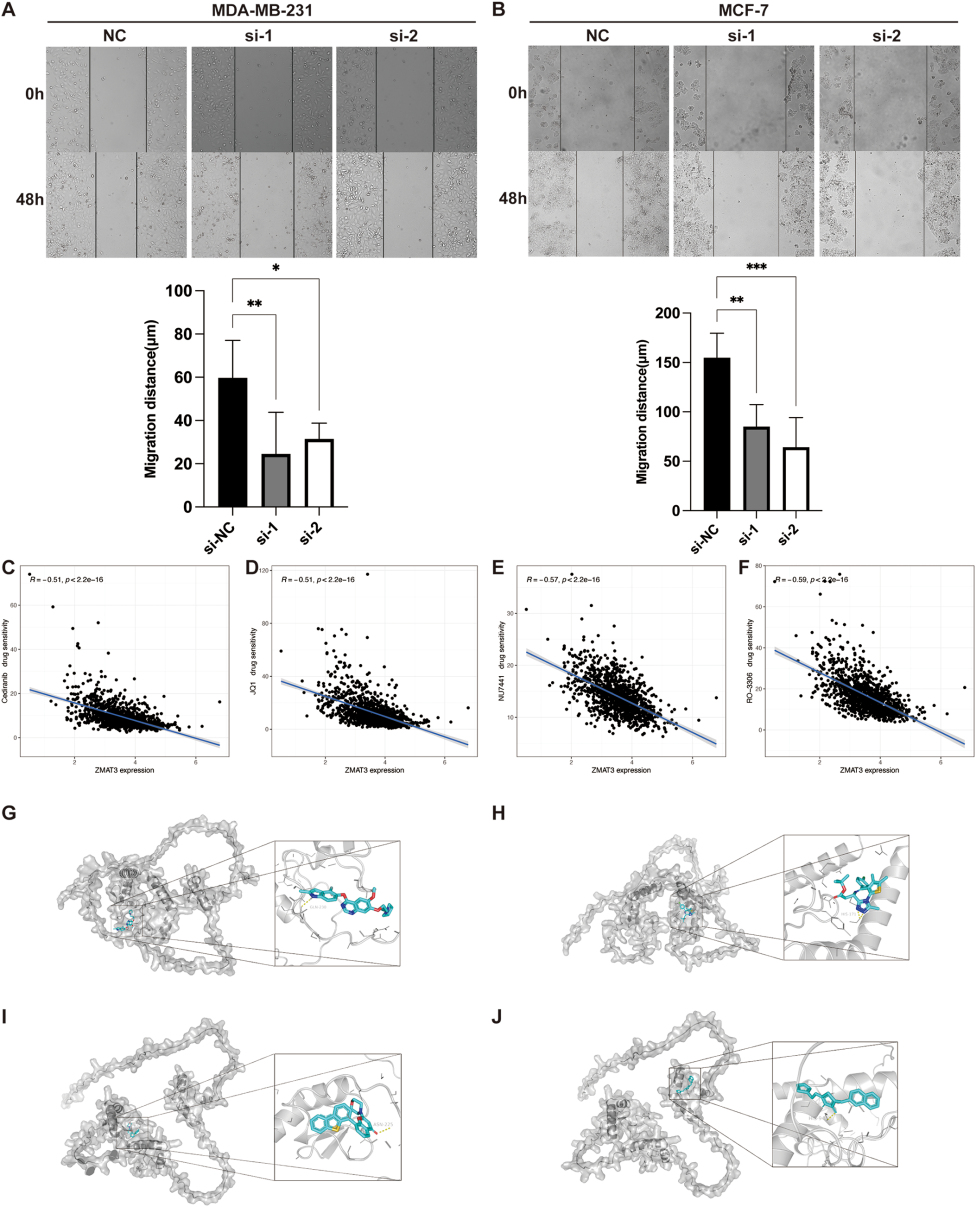
The role of ZMAT3 in breast cancer. **(A, B)** Wound-Healing assay to detect changes in migration ability of MDA-MB-231 and MCF-7 cells after knockdown of ZMAT3. **(C)** Scatterplot of correlation between Cediranib, **(D)** JQ1, **(E)** NU7441, and **(F)** RO-3306 sensitivity and ZMAT3 expression levels. Molecular docking representations of ZMAT3 with **(G)** Cediranib, **(H)** JQ1, **(I)** NU7441, and **(J)** RO-3306. *, P<0.05; **, P<0.01; ***, P<0.001.

Multivariate Cox regression analyses in the TCGA dataset showed that XG and ZMAT3 were independent prognostic risk factors for breast cancer, and ZMAT3 had higher HR values, suggesting that it may be a key gene influencing the prognosis of breast cancer (**Supplementary Figure S5M**). The results of the GSEA enrichment analysis showed that the high-expression ZMAT3 group was predominantly in the Cellular senescence (**Supplementary Figure S5N**). In the TCGA dataset, the expression level of ZMAT3 in tumors was significantly lower than that in adjacent normal tissues (**Supplementary Figure S5O**). In the 32 matched pairs of clinical samples we collected from, the qPCR results were consistent with TCGA (**Figure 12A**). The mRNA expression level of ZMAT3 in human breast cancer cell lines MDA-MB-231 and MCF-7 was significantly lower than that in human breast epithelial cell line MCF-10A (**Figure 12B**). Transfection of siRNAs targeting ZMAT3 was performed in MDA-MB-231 and MCF-7 cells to investigate the regulatory role of ZMAT3 expression in breast cancer cell progression (**Figures 12C, D**).Western blot confirmed that ZMAT3 was successfully knocked down (**Figure 12E**). The proliferation of MDA-MB-231 and MCF-7 cells was significantly inhibited when ZMAT3 was knocked down in CCK-8 assays (**Figures 12F, G**). Additionally, knockdown of ZMAT3 significantly inhibited the invasion and migration ability of breast cancer (**Figures 12H**, **13A, B**). Overall, our findings indicated that ZMAT3 may promote the proliferation and invasion of breast cancer.

Given that the attenuation of ZMAT3 expression diminished the proliferative and invasive propensities of breast cancer cells, we evaluated the sensitivity of ZMAT3 to a spectrum of pharmacological compounds. Our findings reveal a pronounced inverse correlation between ZMAT3 expression and the IC50 values of Cediranib, JQ1, NU7441, and RO-3306, with correlation coefficients all below -0.5 (**Figures 13C–F**). Subsequent molecular docking analyses of ZMAT3 with these agents yielded binding energies of -6.6 kcal/mol for Cediranib, -5.9 kcal/mol for JQ1, -7.9 kcal/mol for NU7441, and -6.7 kcal/mol for RO-3306, respectively, underscoring the potential for efficacious interaction with ZMAT3 (**Figures 13G–J**).

## Discussion

The TGF-β signaling pathway plays a crucial dual role in tumor progression and the shaping of the tumor immune microenvironment. Previous studies have reported that anti-TGF-β/PD-L1 bispecific antibodies, such as YM101 and BiTP, exhibit strong antitumor effects in mouse tumor models (32, 33). However, despite extensive research on individual or several genes within this pathway, our understanding of its overall activity and infiltration characteristics in the TME is still limited. Unraveling the functions of TGF-β signaling pathway activities within the TME could advance our knowledge of the tumor immune microenvironment and guide more precise personalized immunotherapy approaches.

In this study, we first performed a single-cell level analysis to assess the TGF-β signaling pathway activity across various cell types within the breast cancer microenvironment. The highest activity was noted in endothelial cells, fibroblasts, and epithelial cells. At the bulk transcriptome level, we found that the pathway’s activity was significantly higher in normal tissue compared to breast cancer tissue. Through WCGNA analysis, we identified 880 module genes closely associated with TGF-β signaling pathway activity. Crossing these with differentially expressed genes in breast cancer yielded 654 module differential genes.

To establish a prognostic feature linked to the TSP with enhanced predictive accuracy, we compared the C-index of various models using ten machine learning algorithms and 101 algorithm combinations. We ultimately utilized the Lasso method to construct a prognostic feature comprising nine genes: *ENPEP, XG, NACAD, CACNA2D1, ZMAT3, CCND2, SDC1, C11orf24*, and *SGCE*. This feature’s predictive capability was validated in training, internal, and external validation sets. Compared to ten previously published prognostic signatures, our TSPRS demonstrated the highest C-index, indicating superior predictive power. We used the TSPRS to assign risk scores to breast cancer patients, dividing them into high and low-risk groups based on the median risk score. Patients in the low-risk group had a longer OS and higher immune cell infiltration, while the high-risk group exhibited a more potent immune-suppressive TME, leading to reduced patient survival times (34). Both univariate and multivariate COX regression analyses confirmed that TSPRS is an independent prognostic indicator for breast cancer. Additionally, a nomogram integrating TSPRS with clinical pathological characteristics showed excellent performance in predicting 1-year, 5-year, and 7-year OS rates for breast cancer patients.

The molecule PD-L1 on the surface of tumor cells can interact with the PD-1 molecule on T cells’ surface, thereby evading T-cell mediated immune surveillance. By using specific antibodies to block the interaction between PD-1 and PD-L1, the proliferation and cytotoxic functions of T cells can be enhanced, thus playing an anti-tumor role. The levels of immune cell infiltration and PD-L1 expression in TNBC are significantly higher than in other subtypes of breast cancer, indicating that PD-1/PD-L1 inhibitors may have potential therapeutic value in TNBC. Therefore, several clinical studies on ICIs treatment for breast cancer are targeted at TNBC, but other subtypes of breast cancer also possess potential for ICIs treatment. In terms of immunotherapy, our study found that the low-risk group with breast cancer showed higher PD-1 expression levels, greater infiltration of CD8+ T cells and NK cells, and higher IPS, suggesting that these patients might benefit more from ICIs treatment. TSPRS could thus potentially predict immunotherapy response in breast cancer patients. Although data on immunotherapy in breast cancer are scarce, the application of IMV210 indirectly supports TSPRS’s predictive capacity for immunotherapy.

To effectively use TSPRS in managing breast cancer, we suggest applying this model for risk assessment post-diagnosis. By analyzing TSPRS scores, patients can be quickly categorized as high or low risk, guiding treatment decisions. For instance, our study shows that low-risk patients, with higher immune checkpoint expression, may benefit more from single immune checkpoint inhibitor treatments. Conversely, high-risk patients, more responsive to PIK3CA kinase inhibitors, tyrosine kinase inhibitors, estrogen receptor modulators, and standard chemotherapies, and with elevated HAVCR2 levels, might require a combined approach of HAVCR2-targeted immunotherapy and chemotherapy to enhance treatment efficacy.

Focusing on individual genes, our multivariate COX regression analysis identified ZMAT3 and XG as independent prognostic risk factors for breast cancer, with ZMAT3 having the highest HR value. ZMAT3 (Zinc Finger Matrin-Type 3) codes for an RNA-binding protein that contains a zinc finger structure. Despite research indicating that ZMAT3, as a target gene of p53, plays a crucial role in p53-mediated tumor suppression (35), it is recognized as a tumor suppressor factor (36). However, the molecular mechanism through which it operates within cells still needs to be elucidated. For instance, in colorectal cancer, silencing ZMAT3 can promote the proliferation of cancer cells by increasing the inclusion of CD44 variant exons (37). In LUAD, ZMAT3 suppresses tumor growth by inhibiting cell proliferation without inducing cell apoptosis (38). However, research also shows that unlike p53, the absence of ZMAT3 does not affect the development of lymphomas driven by c-Myc or LUAD driven by KRAS mutations (39).

To date, no studies have reported on the role of ZMAT3 in breast cancer. Contrary to previous reports, we found that ZMAT3 is an independent risk factor for poor prognosis in breast cancer, with patients exhibiting high levels of ZMAT3 expression having significantly lower OS compared to those with low expression levels. It has been reported that ZMAT3 is directly transcriptionally controlled by p53 in various cell types (38). In tumors lacking p53, such as breast cancer, ZMAT3 expression is reduced, which is consistent with our experimental results. In the 31 pairs of clinical samples we collected, ZMAT3 expression levels were significantly lower in breast cancer tissues compared to adjacent normal tissues.

p53 primarily induces cell cycle arrest through p21 and 14-3-3σ and apoptosis through target genes such as FAS, PUMA, and NOXA. However, the molecular mechanisms by which cells decide to enter growth arrest or apoptosis following p53 activation are not fully understood. By identifying transcripts affected by ZMAT3 knockdown, researchers found (40) that FAS and 14-3-3σ mRNA are regulated by ZMAT3 in a p53-independent manner. Additionally, ZMAT3 deficiency is associated with increased cell death and reduced cell cycle arrest in response to DNA damage. When we used siRNA transfection to reduce ZMAT3 expression in breast cancer cells, the proliferation and invasion capabilities of these cells were significantly inhibited. GSEA analysis showed that high ZMAT3 expression is associated with enrichment of the cellular senescence pathway. Cellular senescence is a stress response that typically leads to cell cycle arrest. However, in cancer, the senescence mechanism may be exploited by cancer cells to evade apoptosis, which could be one of the mechanisms by which high ZMAT3 expression promotes breast cancer progression.

Inhibiting ZMAT3 may enhance the sensitivity of tumor cells to chemotherapy and radiotherapy by inducing DNA damage. Furthermore, ZMAT3 could serve as a potential target for targeted therapy in breast cancer. Developing inhibitors or small molecule drugs against ZMAT3 could provide new treatment options for breast cancer patients, especially those with high ZMAT3 expression and poor prognosis.

Despite these insights, our study is not without limitations. It is a retrospective study based on public data and requires further validation through large-scale, multi-center prospective studies. To enhance the reliability of TSPRS, we plan to validate this prognostic marker in independent prospective cohorts or clinical trials. Additionally, a more comprehensive integration of clinicopathological features is necessary to fully analyze the clinical value of the risk signature. We recommend designing and implementing prospective studies in future research to evaluate the prognostic predictive ability of TSPRS in different patient populations and to explore its potential application in clinical practice. The role of ZMAT3 in the development and progression of breast cancer also warrants further experimental investigation.

## Data Availability

The raw data supporting the conclusions of this article will be made available by the authors, without undue reservation.

## Supplementary material

Supplementary Figure 1
**(A)**Differential expression analysis of TSPG in breast cancer and adjacent normal tissues. **(B)** Protein-protein interaction network analysis of TSPG in the STRING database. **(C)** Mutation frequency analysis of TSPG in breast cancer. **(D)** Frequencies of CNV gain, loss, and non-CNV among TSPG.

Supplementary Figure 2 Enrichment analysis of TSPRG. **(A)** GO enrichment of TSPRG. **(B)** KEGG enrichment of TSPRG.

Supplementary Figure 3 Kaplan-Meier curves for **(A)** SDC1, **(B)** NACAD, **(C)** ZMAT3, **(D)** CCND2, **(E)** CACNA2D1, **(F)** XG, **(G)** SGCE, **(H)** ENPEP, and **(I)** C11orf24 in the TCGA dataset.

Supplementary Figure 4 The distribution of the risk score, overall survival status of patients and signature gene expression heatmap in **(A–C)** the TCGA training set, **(D–F)** TCGA internal validation set, **(G–I)** TCGA entire set, and **(J–L)** GSE20711. **(M)** Kaplan-Meier curves of OS according to TSPRS in the TCGA entire set. **(N)**ROC curves showing the specificity and sensitivity of TSPRS in predicting 1, 5, and 7-year OS in the TCGA entire set. **(O)** Kaplan-Meier curves of DFS according to TSPRS in the TCGA entire set. **(P)** Kaplan-Meier curves of PFS according to TSPRS in the TCGA entire set. **(R)** Risk coefficients for the nine genes that make up the TSPRS. **(S)** Scatterplot of correlation between TMB and riskScore.

Supplementary Figure 5
**(A)** Heatmap of correlations with clinical features based on TSPRS. Differences in risk scores for **(B)** T, **(C)** N, **(D)** M, and **(E)** stage in high- and low-risk groups. Kaplan-Meier curves for **(F)** M1 and **(G)** HER2+ patients. Differences in **(I)** stromal score, **(J)** immune score, **(K)** estimate score, **(L)** tumor purity in high and low risk groups. **(M)** Forest plot of multivariate Cox regression analysis. **(N)** GSEA analysis of high and low ZMAT3 expression groups. **(O)** Difference in expression of ZMAT3 in TCGA in breast cancer and adjacent normal tissue.
