# Supplementary figures and images for: Unveiling the role of TGF-β signaling pathway in breast cancer prognosis and immunotherapy
Source: Front Oncol. 2024 Nov 27;14:1488137. doi: 10.3389/fonc.2024.1488137 (PMC11631921; doi:10.3389/fonc.2024.1488137)

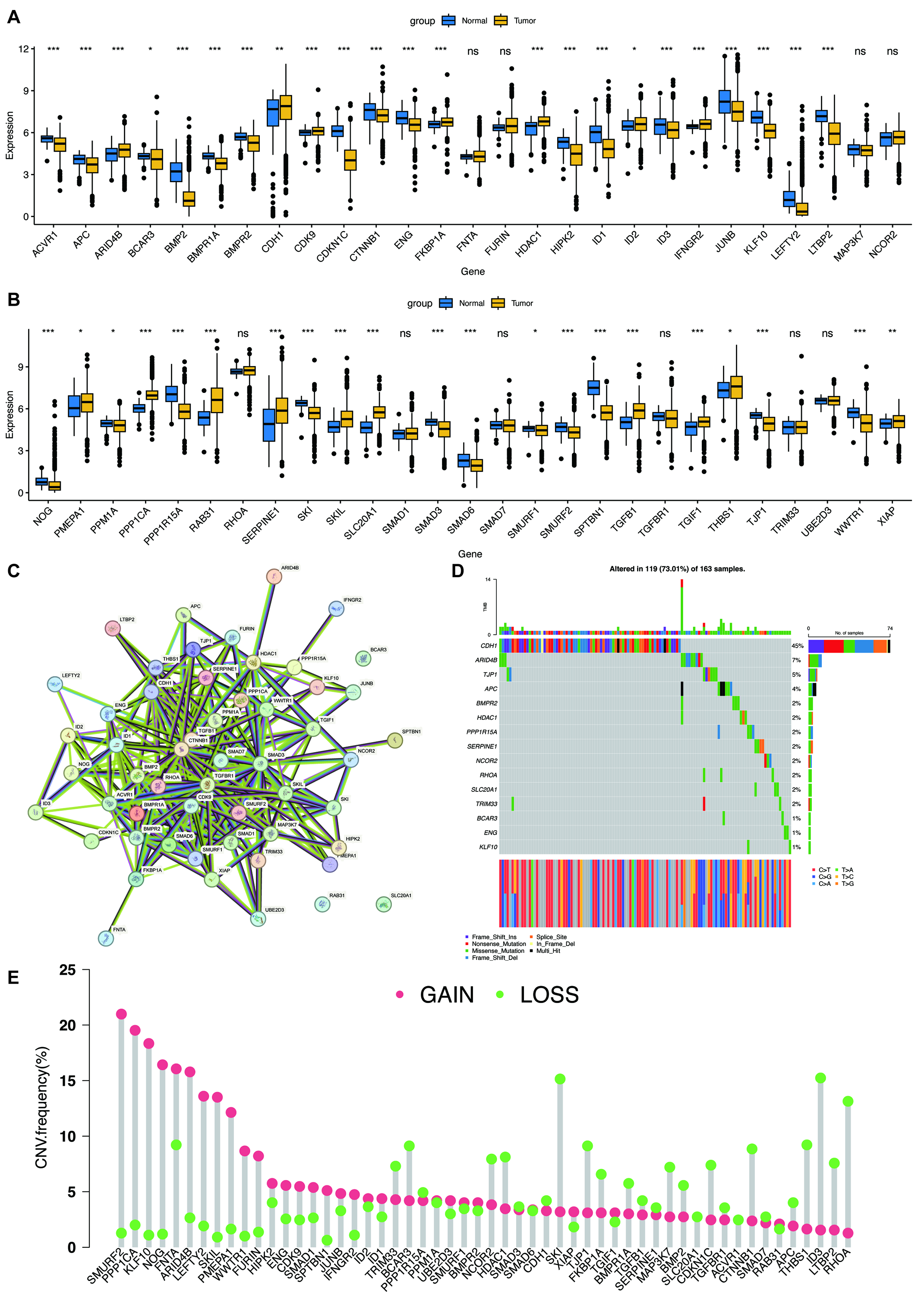

Supplement: Supplementary Figure 1 — (A)Differential expression analysis of TSPG in breast cancer and adjacent normal tissues. (B) Protein-protein interaction network analysis of TSPG in the STRING database. (C) Mutation frequency analysis of TSPG in breast cancer. (D) Frequencies of CNV gain, loss, and non-CNV among TSPG. [file Image1.tif]

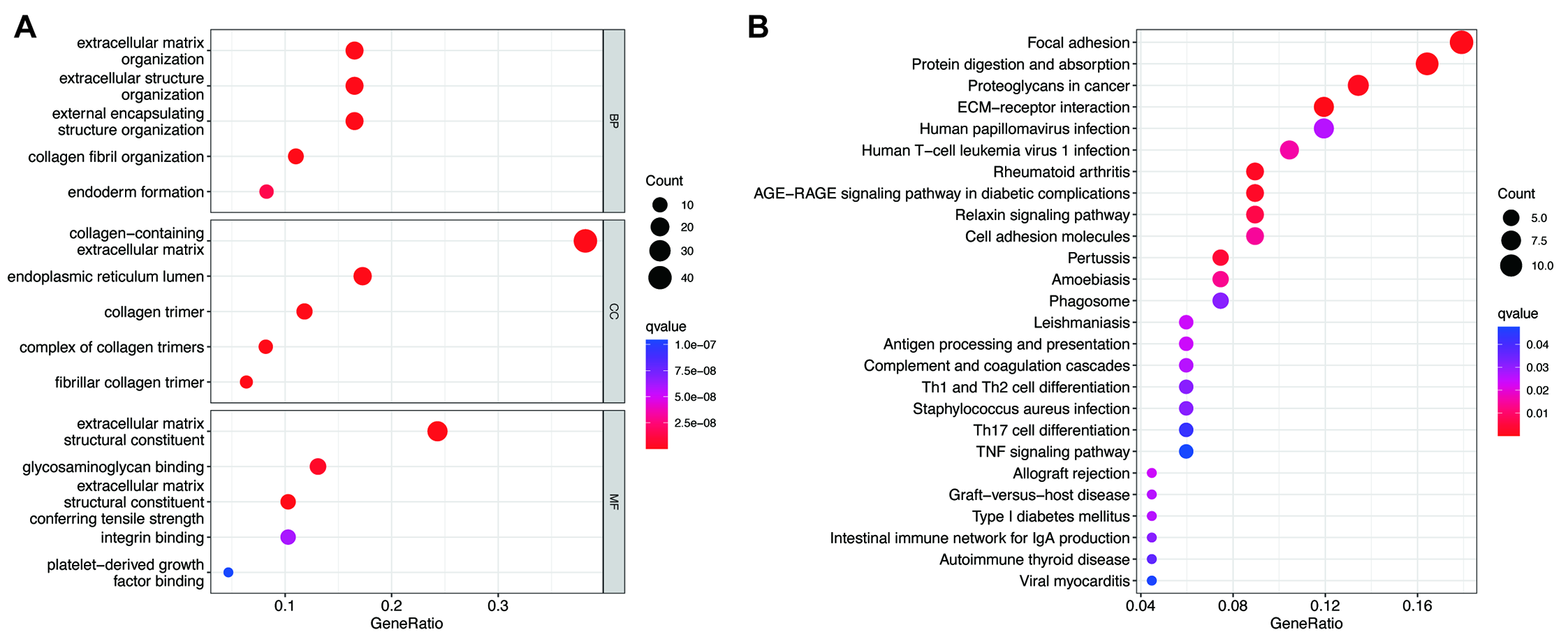

Supplement: Supplementary Figure 2 — Enrichment analysis of TSPRG. (A) GO enrichment of TSPRG. (B) KEGG enrichment of TSPRG. [file Image2.tif]

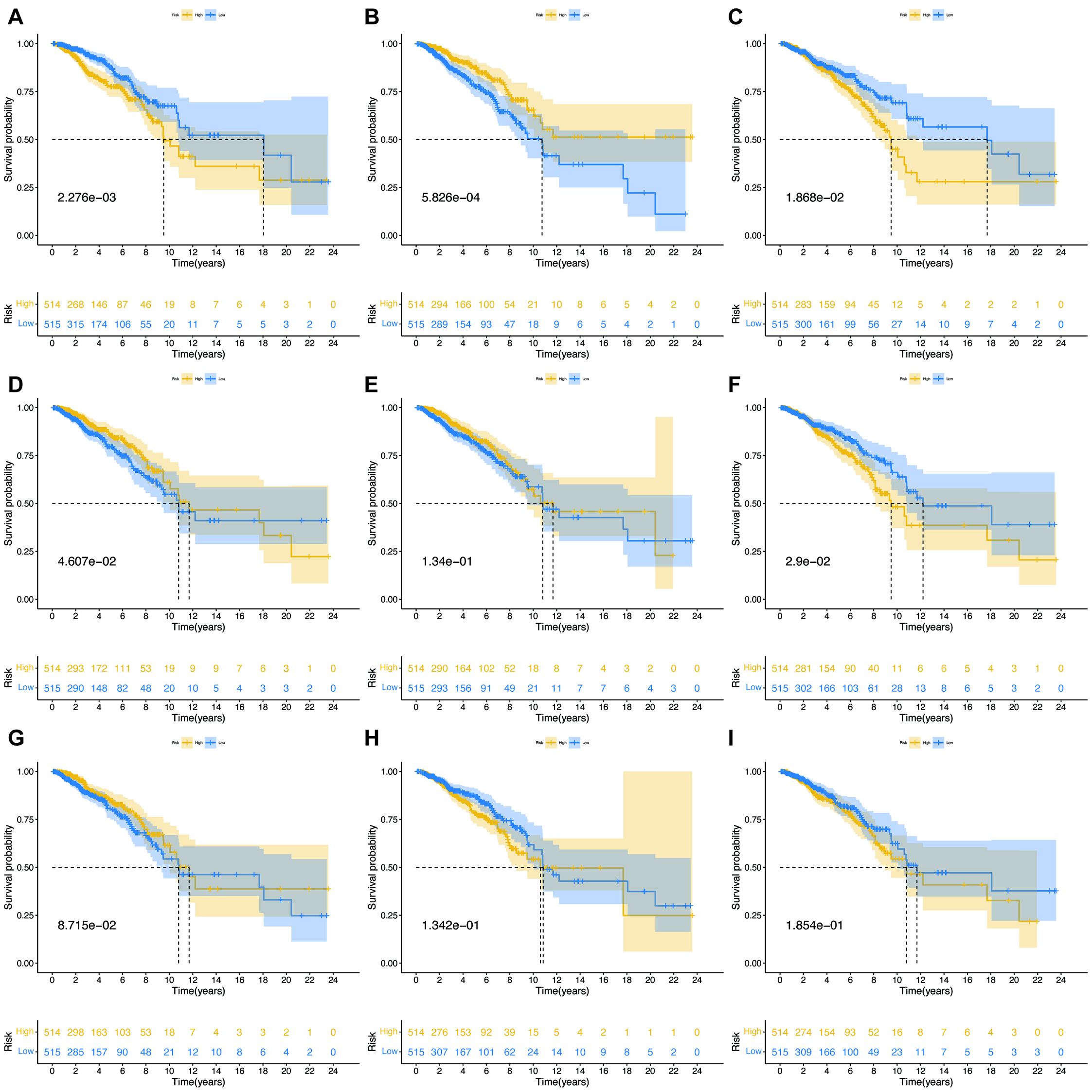

Supplement: Supplementary Figure 3 — Kaplan-Meier curves for (A) SDC1, (B) NACAD, (C) ZMAT3, (D) CCND2, (E) CACNA2D1, (F) XG, (G) SGCE, (H) ENPEP, and (I) C11orf24 in the TCGA dataset. [file Image3.tif]

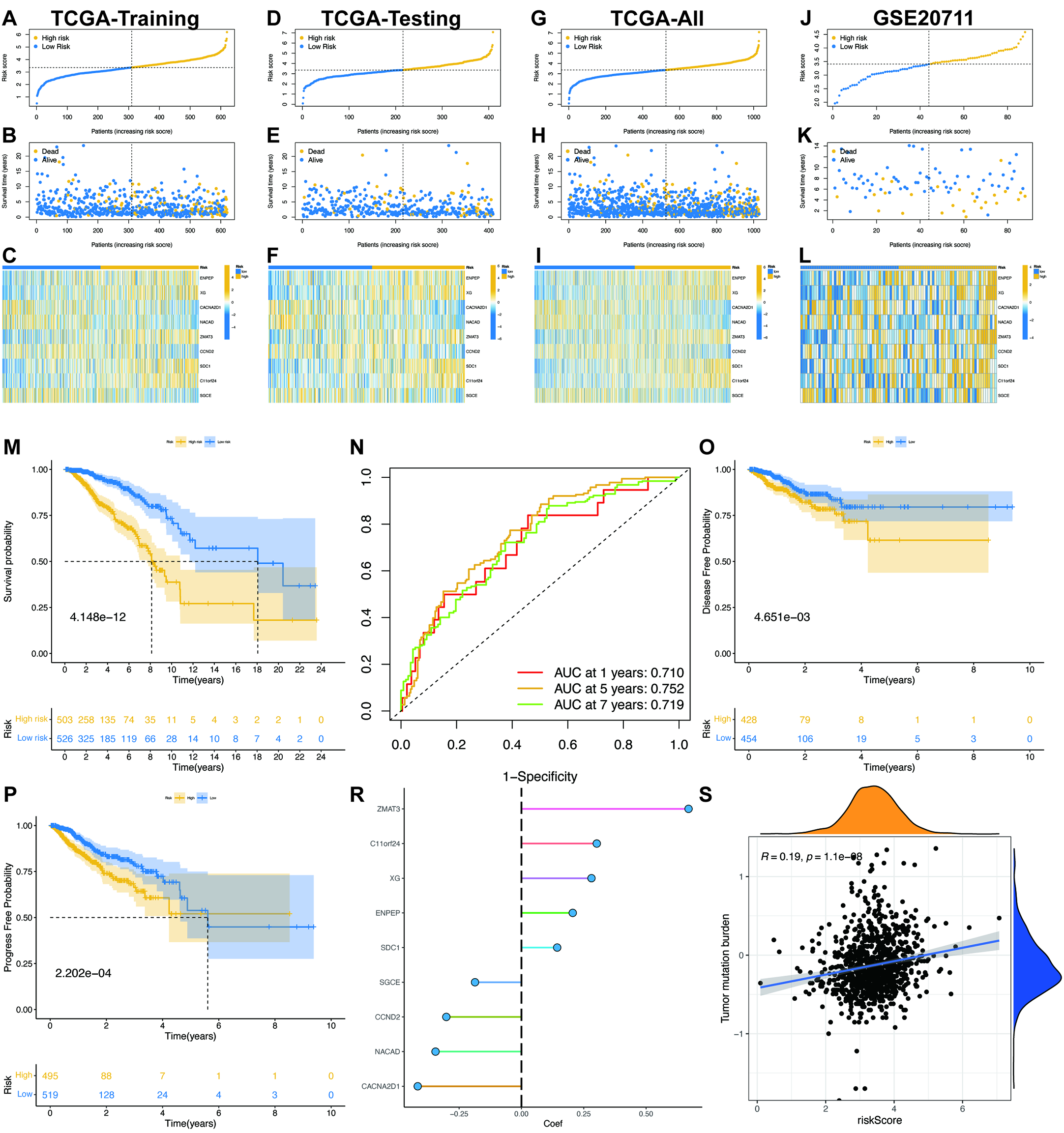

Supplement: Supplementary Figure 4 — The distribution of the risk score, overall survival status of patients and signature gene expression heatmap in (A–C) the TCGA training set, (D–F) TCGA internal validation set, (G–I) TCGA entire set, and (J–L) GSE20711. (M) Kaplan-Meier curves of OS according to TSPRS in the TCGA entire set. (N)ROC curves showing the specificity and sensitivity of TSPRS in predicting 1, 5, and 7-year OS in the TCGA entire set. (O) Kaplan-Meier curves of DFS according to TSPRS in the TCGA entire set. (P) Kaplan-Meier curves of PFS according to TSPRS in the TCGA entire set. (R) Risk coefficients for the nine genes that make up the TSPRS. (S) Scatterplot of correlation between TMB and riskScore. [file Image4.tif]

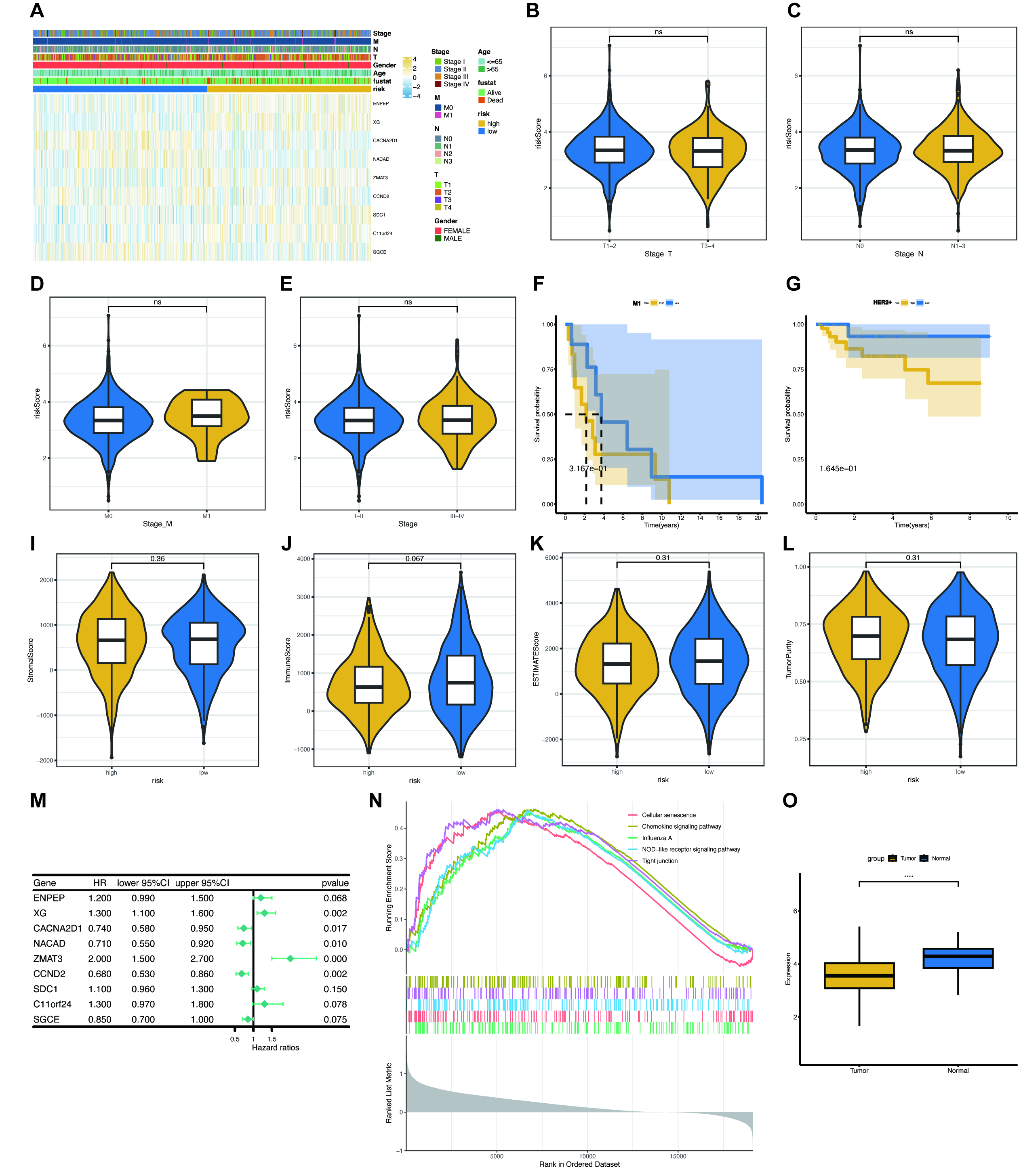

Supplement: Supplementary Figure 5 — (A) Heatmap of correlations with clinical features based on TSPRS. Differences in risk scores for (B) T, (C) N, (D) M, and (E) stage in high- and low-risk groups. Kaplan-Meier curves for (F) M1 and (G) HER2+ patients. Differences in (I) stromal score, (J) immune score, (K) estimate score, (L) tumor purity in high and low risk groups. (M) Forest plot of multivariate Cox regression analysis. (N) GSEA analysis of high and low ZMAT3 expression groups. (O) Difference in expression of ZMAT3 in TCGA in breast cancer and adjacent normal tissue. [file Image5.tif]
